# X-ray structure and characterization of a probiotic *Lactobacillus rhamnosus* Probio-M9 L-rhamnose isomerase

**DOI:** 10.1007/s00253-024-13075-9

**Published:** 2024-03-02

**Authors:** Hiromi Yoshida, Naho Yamamoto, Lin Hai Kurahara, Ken Izumori, Akihide Yoshihara

**Affiliations:** 1 Department of Basic Life Science, Faculty of Medicine, Kagawa University, 1750-1 Ikenobe, Miki-Cho, Kita-Gun, Kagawa 761-0793 Japan; 2 International Institute of Rare Sugar Research and Education, Kagawa University, Takamatsu, Kagawa Japan; 3 Faculty of Agriculture, Kagawa University, 2393 Ikenobe, Miki, Kagawa 761-0795 Japan; 4 Department of Cardiovascular Physiology, Faculty of Medicine, Kagawa University, 1750-1 Ikenobe, Miki-Cho, Kita-Gun, Kagawa 761-0793 Japan

**Keywords:** Crystal structure, D-allose, D-allulose, *Lactobacillus rhamnosus* Probio-M9, L-rhamnose isomerase, Rare sugar

## Abstract

**Abstract:**

A recombinant L-rhamnose isomerase (L-RhI) from probiotic *Lactobacillus rhamnosus* Probio-M9 (*L. rhamnosus* Probio-M9) was expressed. *L. rhamnosus* Probio-M9 was isolated from human colostrum and identified as a probiotic lactic acid bacterium, which can grow using L-rhamnose. L-RhI is one of the enzymes involved in L-rhamnose metabolism and catalyzes the reversible isomerization between L-rhamnose and L-rhamnulose. Some L-RhIs were reported to catalyze isomerization not only between L-rhamnose and L-rhamnulose but also between D-allulose and D-allose, which are known as rare sugars. Those L-RhIs are attractive enzymes for rare sugar production and have the potential to be further improved by enzyme engineering; however, the known crystal structures of L-RhIs recognizing rare sugars are limited. In addition, the optimum pH levels of most reported L-RhIs are basic rather than neutral, and such a basic condition causes non-enzymatic aldose-ketose isomerization, resulting in unexpected by-products. Herein, we report the crystal structures of *L. rhamnosus* Probio-M9 L-RhI (LrL-RhI) in complexes with L-rhamnose, D-allulose, and D-allose, which show enzyme activity toward L-rhamnose, D-allulose, and D-allose in acidic conditions, though the activity toward D-allose was low. In the complex with L-rhamnose, L-rhamnopyranose was found in the catalytic site, showing favorable recognition for catalysis. In the complex with D-allulose, D-allulofuranose and ring-opened D-allulose were observed in the catalytic site. However, bound D-allose in the pyranose form was found in the catalytic site of the complex with D-allose, which was unfavorable for recognition, like an inhibition mode. The structure of the complex may explain the low activity toward D-allose.

**Key points:**

• *Crystal structures of LrL-RhI in complexes with substrates were determined.*

• *LrL-RhI exhibits enzyme activity toward L-rhamnose, D-allulose, and D-allose.*

• *The LrL-RhI is active in acidic conditions.*

**Supplementary Information:**

The online version contains supplementary material available at 10.1007/s00253-024-13075-9.

## Introduction

*Lactobacilli* and *Bifidobacteria* are representative intestinal microorganisms, and some species and strains are living probiotic bacteria that confer health benefits on the hosts (Hill et al. 2014). For example, *Bifidobacterium longum* subsp. *infantis* was reported to consume human milk oligosaccharides and grow efficiently (Marcobal et al. 2010), but this is not common for all bifidobacteria (LoCascio et al. 2009). Human breast milk contains essential nutrients for infants and is also known for the presence of bacteria. Since reports on the bacterial diversity of human milk (Heikkilä and Saris 2003; Martín et al. 2003), numerous studies of the bacterial communities in human milk have been reported (Kozak et al. 2015; Ruiz et al. 2019; Liu et al. 2020). From breast milk, facultative anaerobes such as *Staphylococcus*, *Streptococc*us, *Enterococcus*, *Lactobacillus*, and *Bifidobacterium* have been isolated (Heikkilä and Saris 2003; Sakwinska et al. 2016). More recently, Liu et al. (2020) isolated probiotic lactic acid bacteria and bifidobacteria from human colostrum. They characterized *L. rhamnosus* Probio-M9 and *Bifidobacterium lactis* Probio-M8 and reported them as probiotic bacteria.

**Fig. 1:**
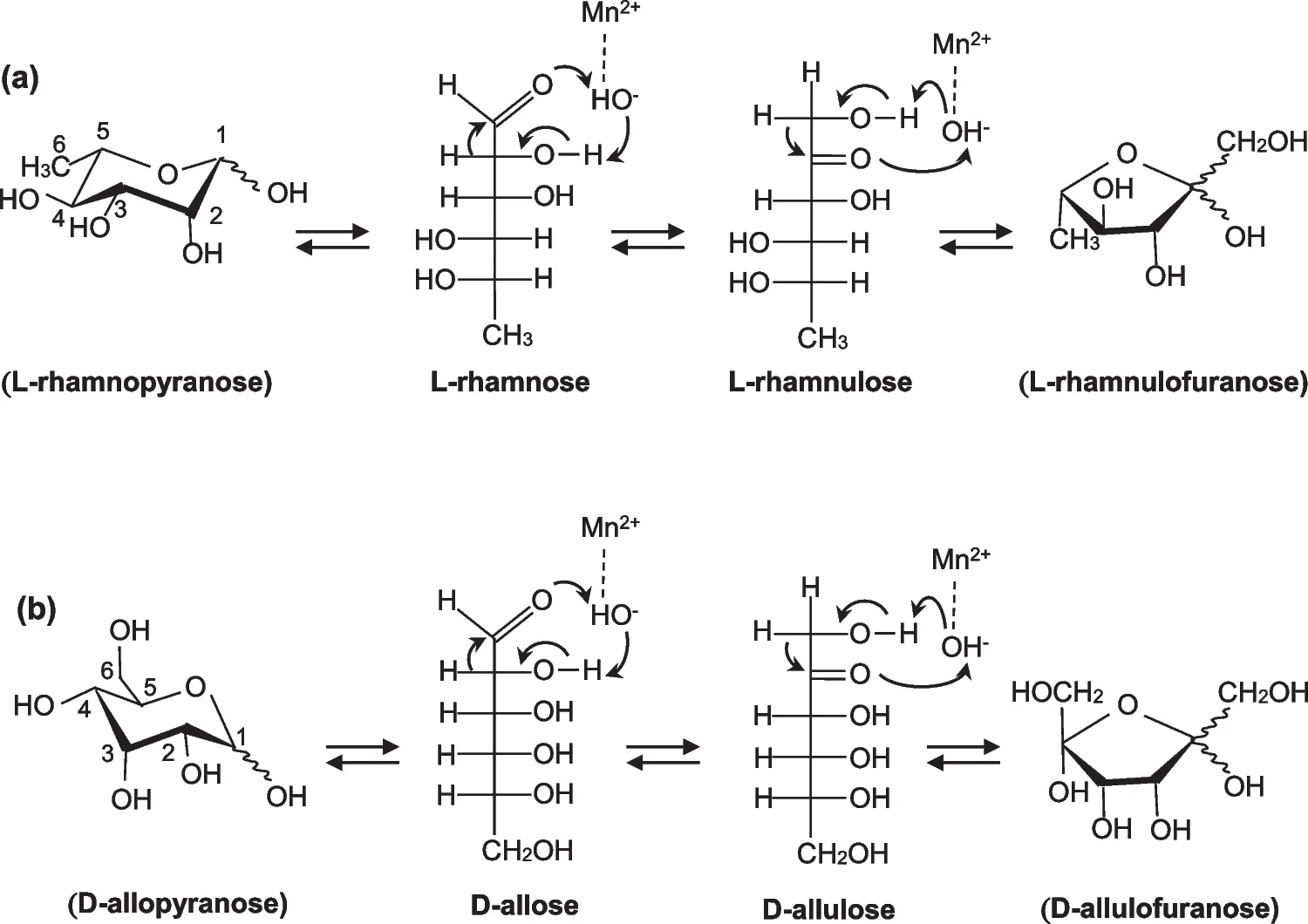
Proposed aldose-ketose isomerization catalyzed by L-RhIs based on the metal-mediated hydride-shift mechanism. **a** Chemical reaction between L-rhamnose and L-rhamnulose with sugar-ring form, catalyzed by L-RhIs from *Escherichia coli* (Korndörfer et al. 2000) and *Pseudomonas stutzeri* (Yoshida et al. 2007, 2010b, 2012). Mn^2+^ represents a catalytic metal ion. **b** Chemical reaction between D-allose and D-allulose with sugar-ring form, catalyzed by *P. stutzeri* L-RhI. Mn^2+^ represents a catalytic metal ion (Yoshida et al. 2007, 2010b, 2012)

*L. rhamnosus* Probio-M9 can grow using L-rhamnose and under acidic conditions. From the genome analysis of the Probio-M9 strain (Ruibo et al. 2023), several genes related to metabolized L-rhamnose, putative L-rhamnose mutarotase, putative L-RhI, putative L-rhamnulose kinase, and putative L-rhamnulose-1-phosphate aldolase have been identified; these were also reported in *Escherichia coli* (*E. coli*). (Wilson and Ajl 1957; Power 1967; Moralejo et al. 1993). Although these are putative enzymes, we are interested in expressing and characterizing a synthesized gene encoding a promising enzyme derived from such probiotic bacteria. In this study, a putative L-RhI from *L. rhamnosus* Probio-M9 (LrL-RhI) was selected as a candidate for characterization and structure analysis. L-RhI is an enzyme that can catalyze the isomerization between L-rhamnose and L-rhamnulose (Fig. 1a) and is involved in L-rhamnose metabolism in *E. coli*, as mentioned above.

The existence of L-RhI-derived *Lactobacillus* has been reported in *Lactobacillus plantarum* (Domagk and Zech 1963). Furthermore, L-RhI from *Pseudomonas stutzeri* (PsL-RhI) was reported to catalyze isomerization not only between L-rhamnose and L-rhamnulose but also between D-allulose and D-allose (Fig. 1b) (Bhuiyan et al. 1997; Leang et al. 2004a, b). D-Allulose and D-allose are rare sugars that exhibit physiological effects, suppression of postprandial blood-sugar elevation, accumulation of visceral fat by D-allulose (Hayashi, et al. 2010; Iida et al. 2013), antioxidation effect, and the growth inhibition of cancer cells by D-allose (Sun et al. 2006; Noguchi et al. 2016; Shintani et al. 2020). As the amount of each rare sugar is extremely low in nature, potential enzymes for rare sugar production are widely screened and studied. A number of D-allulose 3-epimerases from various microorganisms have been reported that can produce D-allulose from abundant D-fructose by epimerization at the C3 position. Using the produced D-allulose, D-allose production is expected to be achieved via ketose-aldose isomerization by L-RhI in future experiments.

To date, a variety of L-RhIs have been reported and characterized (*Pseudomonas stutzeri*, Leang et al. 2004b; *Bacillus pallidus*, Poonperm et al. 2007; *Thermoanaerobacterium saccharolyticum*, Lin et al. 2010; *Thermotoga maritima*, Park et al. 2010; *Caldicellulosiruptor saccharolyticus*, Lin et al. 2011; *Bacillus halodurans*, Prabhu et al. 2011; *Mesorhizobium loti*, Takata et al. 2011; *Dictyoglomus turgidum*, Kim et al. 2013; *Bacillus subtilis*, Bai et al. 2015; Park 2014; and *Clostridium stercorarium*, Seo et al. 2018). These show broad substrate specificity and have the potential to produce various rare sugars. However, the enzyme activity of L-RhIs on D-allose is relatively low for industrial usage (*Pseudomonas stutzeri* L-RhI, 7.5 U/mg, Leang et al. 2004b; *Mesorhizobium loti* L-RhI, 3.0 U/mg, Takata et al. 2011; *Bacillus pallidus* L-RhI, 2.6 U/mg, Poonperm et al. 2007; *Caldicellulosiruptor obsidiansis*, L-RhI, 13.7 U/mg, Chen et al. 2018a; *Thermobacillus composti* L-RhI, 1.7 U/mg; *Thermotoga maritima* L-RhI, 6.7 U/mg, Park et al. 2010; *Thermoanaerobacterium saccharolyticum* L-RhI, 5.7 U/mg, Lin et al. 2010). Thus, several studies seeking to alter the substrate specificity or improve activity toward D-allose have been conducted (Tseng et al. 2022; Chen et al. 2018b; Duan et al. 2023).

Despite the existence of L-RhIs exhibiting enzymatic activity toward D-allose, the known crystal structures of L-RhIs are limited. Available structures of L-RhIs in the Protein Data Bank (PDB) are only from *Escherichia coli* (L-rhamnose isomerase from *Escherichia* *coli *(EcL-RhI), 419 amino acid residues, 50% sequence identity with LrL-RhI) (Korndörfer et al. 2000), *Pseudomonas stutzeri* (PsL-RhI, 430 a.a., 12.2% identity) (Yoshida et al. 2007), and *Bacillus halodurans* (currently known as *Halalkalibacterium halodurans*, L-rhamnose isomerase from *Bacillus halodurans *(BhL-RhI), 418 a.a., 50% identity) (Prabhu et al. 2014). In addition, the optimum pH of most reported L-RhIs is basic, which causes non-enzymatic aldose-ketose isomerization of the substrate and product, resulting in unexpected by-products such as D-altrose. Therefore, L-RhIs that are active under acidic conditions may be promising enzymes for the production of various rare sugars, including D-allose. Herein, we report the crystal structure of LrL-RhI, which shows enzyme activity toward D-allulose and D-allose, and its optimum pH for activity is acidic (pH 4.5–5.5). Furthermore, we report the structure of the complex with D-allose, revealing an undesirable binding mode in the catalytic site, which may explain the low enzymatic activity toward D-allose.

## Materials and methods

### Cloning and expression

The amino acid sequence of the putative LrL-RhI (426 amino acid residues, 48707 Da) is the same as that of provisional L-RhI from *Lacticaseibacillus rhamnosus* (NCBI, WP_019728362.1). Using the synthesized gene encoding the putative LrL-RhI (Supplemental Fig. S1), an expression vector was constructed. The synthesized gene was amplified using the forward (5′- GAGAAATTAACCATGGTAAAACCTGAAGAGGTGG-3′) and reverse (5’- GATGGTGATGAGATCCGTGCACCAGCTTTGCAT-3′) primers and then mixed with the pQE60 digested by *Nco*I and *Bgl*II for the in-fusion reaction using the In-Fusion HD Cloning Kit (TaKaRa Bio Inc., Shiga, Japan). The resultant construct contains His-tag at the C-terminus of LrL-RhI, and the recombinant LrL-RhI was expressed in *E. coli* JM109.

### Protein preparation

Recombinant LrL-RhI expressed in *E. coli* cells were grown at 37 ℃ in a 2 × YT medium containing 100 μg/ml ampicillin until the cell density at 600 mm (OD600) reached 0.4–0.5 and then was cultivated at 25 ℃ overnight after the addition of 0.3 mM isopropyl-β-D-thiogalactopyranoside (IPTG) and 1 mM MnCl_2_. Harvested cells were disrupted in the buffer (50 mM NaH_2_PO_4_, 300 mM NaCl (pH 8.0)) by sonication, and the cell debris was removed by centrifugation (20,400 g, 30 min, 4 ℃). The supernatant was applied onto an affinity column (HisTrap HP 5 ml, Cytiva, Tokyo, Japan). Recombinant LrL-RhI was eluted using a linear gradient of 20–500 mM imidazole with the same sonication buffer and dialyzed against a 5 mM Tris–HCl buffer (pH 8.0) overnight. The purified C-terminal His-tagged LrL-RhI protein solution was concentrated at 5.6 mg/ml using an Amicon Ultra-15 30 kDa Ultracel (Millipore, Billerica, MA, USA) for crystallization. For enzyme characterization, a super broth medium was used for growing the recombinant cells instead of a 2×YT medium, and this was cultivated at 25 ℃ for 6 h after the addition of 1.0 mM IPTG. The enzyme was purified in the same manner and dialyzed against a 20 mM Tris–HCl buffer (pH 7.5).

### Characterization of LrL-RhI

Next, the enzyme activity, pH effects, temperature effects, metal ion effects, stability, and substrate specificity of LrL-RhI were investigated.

The enzymatic activity of LrL-RhI was measured by a cysteine-carbazole assay, detecting the amount of ketose produced using a calorimetric method (Dische and Borenfreund 1950). Briefly, a reaction mixture consisting of the purified enzyme solution in the buffer (a 35 mM sodium phosphate buffer (pH 7.0)) containing 1 mM MnCl_2_ and 5 mM L-rhamnose was incubated at 50 ℃ for 10 min. The reaction was terminated by the addition of trichloroacetic acid. The amount of L-rhamnulose that formed was measured at 540 nm after inducing color development by the addition of cysteine, sulfuric acid, and carbazole and incubating the mixture at 35 ℃ for 20 min (Leang et al. 2004a; Yoshida et al. 2010a). All measurements were performed in triplicate. One unit of enzyme activity was defined as the amount of enzyme required to produce 1 μmol of ketose/min.

To investigate the optimum pH of LrL-RhI, the enzyme activity was measured using the following buffers: a glycine–HCl buffer for pH 2.5–3.6, an acetate buffer for pH 3.6–5.5, a sodium phosphate buffer for pH 5.5–8.0, a Tris–HCl buffer for pH 8.0–9.0, and a glycine–NaOH buffer for pH 9.0–10.0. To investigate the pH stability, LrL-RhI was incubated at 4 °C for 24 h in 20 mM of each buffer, and the remaining activity was measured using a sodium phosphate buffer (pH 5.5).

The optimum reaction temperature was investigated by measuring the enzyme activity after incubation at each temperature (30, 40, 50, 60, 70, and 80 ℃) for 10 min using a sodium phosphate buffer (pH 5.5). To investigate the effect of temperature on enzyme stability, LrL-RhI was incubated at each temperature (4, 30, 40, 50, 60, 70, and 80 ℃) for 10 min and then reacted at 70 ℃ in a 35 mM sodium phosphate buffer (pH 5.5) containing 5 mM L-rhamnose and 1 mM MnCl_2_, and the relative activity was determined.

The effects of metal ions on LrL-RhI were tested after 10 mM EDTA treatment to remove bound metal ions from the purified sample. The EDTA-treated sample was dialyzed against a buffer (20 mM Tris–HCl, pH 7.5) to remove EDTA, and the enzyme activity was measured using a sodium phosphate buffer (pH 5.5) containing 5 mM L-rhamnose and 1 mM of each metal ion (MnCl_2_, CoCl_2_, MgCl_2_, CuSO_4_, CaCl_2_, FeSO_4_, NiCl_2_, and ZnSO_4_).

After the preferable metal ion was determined, the substrate specificity of LrL-RhI was investigated using a 35 mM sodium phosphate buffer (pH 5.5) containing 5 mM of each aldose (D-ribose, L-lyxose, L-rhamnose, D-allose, L-mannose, D-gulose, and L-talose) and 1 mM CoCl_2_. The enzyme reaction was performed at 60 ℃, taking into consideration the thermal stability of LrL-RhI.

### X-ray crystallography

The crystals of LrL-RhI were grown in a droplet containing 0.8 μl of protein solution (5.6 mg/ml in 5 mM Tris–HCl (pH 8.0)) and 0.8 μl of reservoir solution (0.1 M MES (pH 6.5), 15% (v/v) PEG550MME) against 50 μl of reservoir solution by sitting-drop vapor-diffusion at 20 ℃. A crystal was directly flash-cooled in a stream of nitrogen gas at − 173 ℃. To determine the structures of the complexes, each crystal was soaked in a solution containing 30% (w/v) L-rhamnose, D-allulose, or D-allose before flash-cooling. Co-crystallization of the protein with the substrates was not performed. X-ray diffraction data were collected using the PILATUS3 S2M PAD detector on the PF-AR NW12A beamline at the KEK facility (Tsukuba, Japan). Diffraction data were processed using the XDS (Kabsch 2010) and CCP4 program suite (Winn et al. 2011). The initial phases of LrL-RhI were obtained by molecular replacement using the MOLREP program (Vagin and Teplyakov 1997, 2010) with the structure of L-RhI from *E. coli* with L-rhamnitol (PDB code: 1de5) as a probe model. Further model building was performed using the Coot program (Emsley et al. 2010), and the structure was refined using the Refmac5 program (Murshudov et al. 1997, 2011). Data collection and refinement statistics are summarized in Table 1. The refined structures were deposited in the PDB as 8JQ3 (LrL-RhI), 8JQ4 (LrL-RhI in complex with L-rhamnose: LrL-RhI/L-rhamnose), 8JQ5 (LrL-RhI in complex with D-allulose: LrL-RhI/D-allulose), and 8JQ6 (LrL-RhI in complex with D-allose: LrL-RhI/D-allose). Figures 4, 5, S2, and S3 were drawn using the PyMol program (Schrödinger, LLC., New York, USA).

**Table 1:** Data collection and refinement statistics

Data collection	*L. rhamnosus* L-RhI	*L. rhamnosus* L-RhI/L-rhamnose	*L. rhamnosus* L-RhI/D-allulose	*L. rhamnosus* L-RhI/D-allose
Beamline	PF-AR NW12A	PF-AR NW12A	PF-AR NW12A	PF-AR NW12A
Temperature (K)	100	100	100	100
Wavelength (Å)	1.000	1.000	1.000	1.000
Space group	*P*2_1_2_1_2_1_	*P*2_1_2_1_2_1_	*P*2_1_2_1_2_1_	*P*2_1_2_1_2_1_
Unit cell parameters (Å)	*a* = 89.70	*a* = 90.12	*a* = 89.89	*a* = 89.93
	*b* = 139.94	*b* = 140.79	*b* = 139.45	*b* = 140.06
	*c* = 147.31	*c* = 147.35	*c* = 146.83	*c* = 147.47
Resolution range (Å)	50.00–1.90 (1.95–1.90)	46.93–1.61 (1.64–1.61)	48.94–1.73 (1.76–1.73)	49.16–1.71 (1.74–1.71)
No. of measured reflections	1,971,992	3,204,608	2,569,188	2,640,827
No. of unique reflections	146,232 (10,740)	241,662 (11,906)	192,191 (9,422)	200,687 (9,801)
Redundancy	13.5 (14.0)	13.3 (13.4)	13.4 (13.5)	13.2 (12.7)
Completeness (%)	100.0 (100.0)	100.0 (100.0)	100.0 (100.0)	100.0 (99.6)
Mean *I*_*o*_/σ(*I*_*o*_)	22.9 (4.2)	16.7 (2.1)	17.5 (2.2)	12.6 (2.1)
*R*_merge_^§^ (%)	8.6 (68.9)	11.5 (140.2)	11.8 (141.4)	14.8 (133.8)
CC_1/2_	0.999 (0.892)	0.999 (0.711)	0.999 (0.722)	0.998 (0.700)
Refinement				
Resolution range (Å)	44.51–1.90 (1.95–1.90)	45.10–1.61 (1.65–1.61)	46.23–1.73 (1.78–1.73)	46.43–1.71 (1.75–1.71)
No. of reflections, working set	138,900 (10,205)	227,211 (16,841)	182,641 (13,387)	190,503 (13,921)
No. of reflections, test set	7237 (522)	11,882 (879)	9442 (685)	10,079 (730)
Completeness (%)	100.0 (100.0)	99.0 (100.0)	100.0 (100.0)	100.0 (99.8)
*R*_work_† (%)	16.3 (20.2)	16.7 (19.8)	14.2 (19.7)	14.2 (20.8)
*R*_free_^‡^ (%)	21.8 (29.4)	20.8 (26.6)	18.7 (26.6)	18.7 (26.1)
R.m.s.d. bond length (Å)	0.004	0.003	0.005	0.005
R.m.s.d. bond angles (°)	1.18	1.10	1.26	1.22
Ramachandran plot				
Preferred region (%)	94.1	94.7	94.2	95.1
Allowed region (%)	5.3	9.8	5.0	4.4
B-factor (Å^2^)				
Protein	27.4	20.3	22.9	20.5
Mn	31.2	29.6	31.7	33.5
Ligand		23.9 (L-rhamnose, catalytic site) 33.5 (L-rhamnose, surface)	32.4 (D-allulose, catalytic site) 54.7 (D-allulose, surface)	35.9 (D-allose, catalytic site) 36.8 (D-allose, surface)
Water	29.6	31.0	24.8	23.7
PDB ID	8JQ3	8JQ4	8JQ5	8JQ6

Values in parentheses are from the high-resolution bin

^§^*R*_merge_ = Σ_*hkl*_ Σ_*i*_ [|*I*_*i*_(*hkl*) −  < *I*(*hkl*) >|/ Σ_*hkl*_ Σ_*i*_* I*_*i*_(*hkl*)], where *I*_*i*_(*hkl*) is the intensity value of the *i*th measurement of reflection *hkl* and < *I*(*hkl*) > is the mean value of *I*_*i*_(*hkl*) for all *i* measurements. †*R*_work_ = Σ_*hkl*_ ||*F*_obs_| −|*F*_calc_||/ Σ_*hkl*_ |*F*_obs_|, where *F*_obs_ and *F*_calc_ are the observed and calculated structure factors, respectively. ‡*R*_free_ is the free *R*_work_ for the 5% of reflections that were excluded from the refinement

## Results

### Purification and properties of LrL-RhI

The recombinant LrL-RhI was successfully overexpressed and purified with a specific activity of 1.17 U/mg protein using L-rhamnose as the substrate. The purity of the enzyme was confirmed by SDS-PAGE (Supplemental Fig. S2), revealing a single band with an estimated molecular mass of 49 kD.

**Fig. 2:**
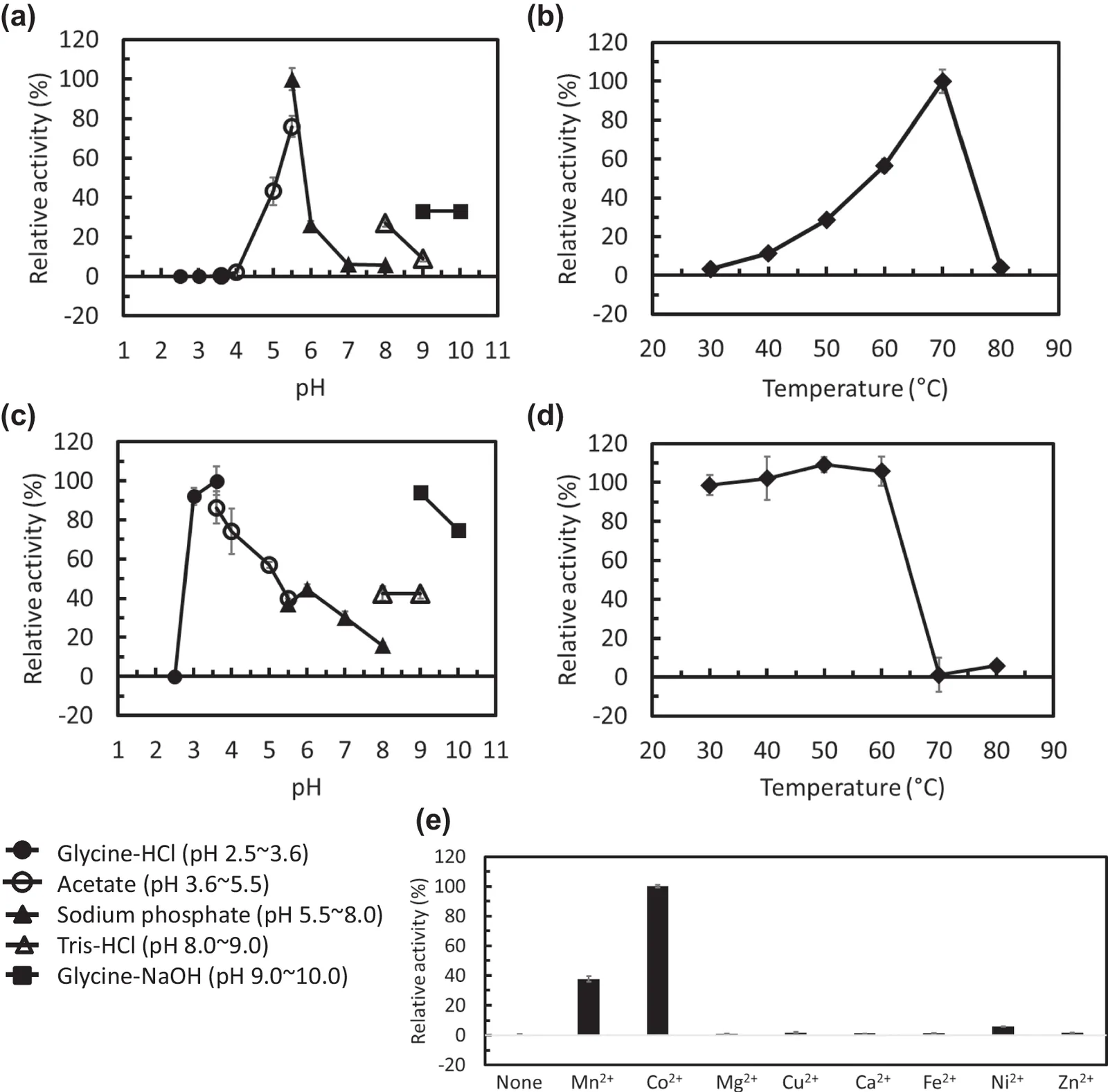
Enzyme properties of recombinant LrL-RhI. Effects of **a** pH on the enzyme activity, **b** temperature on the enzyme activity, **c** pH on the enzyme stability, **d** temperature on the enzyme stability, and **e** metal ions on the enzyme activity. To investigate the effects of pH (**a**), enzyme activity was measured using the following buffers: glycine–HCl buffer (pH 2.5–3.6, circles), acetate buffer (pH 3.6–5.5, open circles), sodium phosphate buffer (pH 5.5–8.0, triangles), Tris–HCl buffer (pH 8.0–9.0, open triangles), and glycine–NaOH buffer (pH 9.0–10.0, squares). The optimum pH was determined to be 5.5. To investigate the effect of temperature (**b**), enzyme activity was measured after incubation at each temperature for 10 min using a sodium phosphate buffer (pH 5.5). The highest enzyme activity was observed at 70 ℃. To investigate the pH stability (**c**), the enzyme was incubated at 4 ℃ for 24 h in 20 mM of each buffer, and the remaining activity was measured using a sodium phosphate buffer (pH 5.5), and the enzyme reaction was performed at 70 ℃ for 10 min. To investigate thermal stability (**d**), LrL-RhI was incubated at each temperature (4, 30, 40, 50, 60, 70, and 80 ℃) for 10 min, then reacted at 70 ℃ in a 35 mM sodium phosphate buffer (pH 5.5) containing 5 mM L-rhamnose and 1 mM MnCl_2_, and the relative activity was determined. To investigate metal specificity (**e**), the enzyme activity was measured using a sodium phosphate buffer (pH 5.5) containing 5 mM L-rhamnose and 1 mM of each metal ion. Each measurement was performed in triplicate

**Fig. 3:**
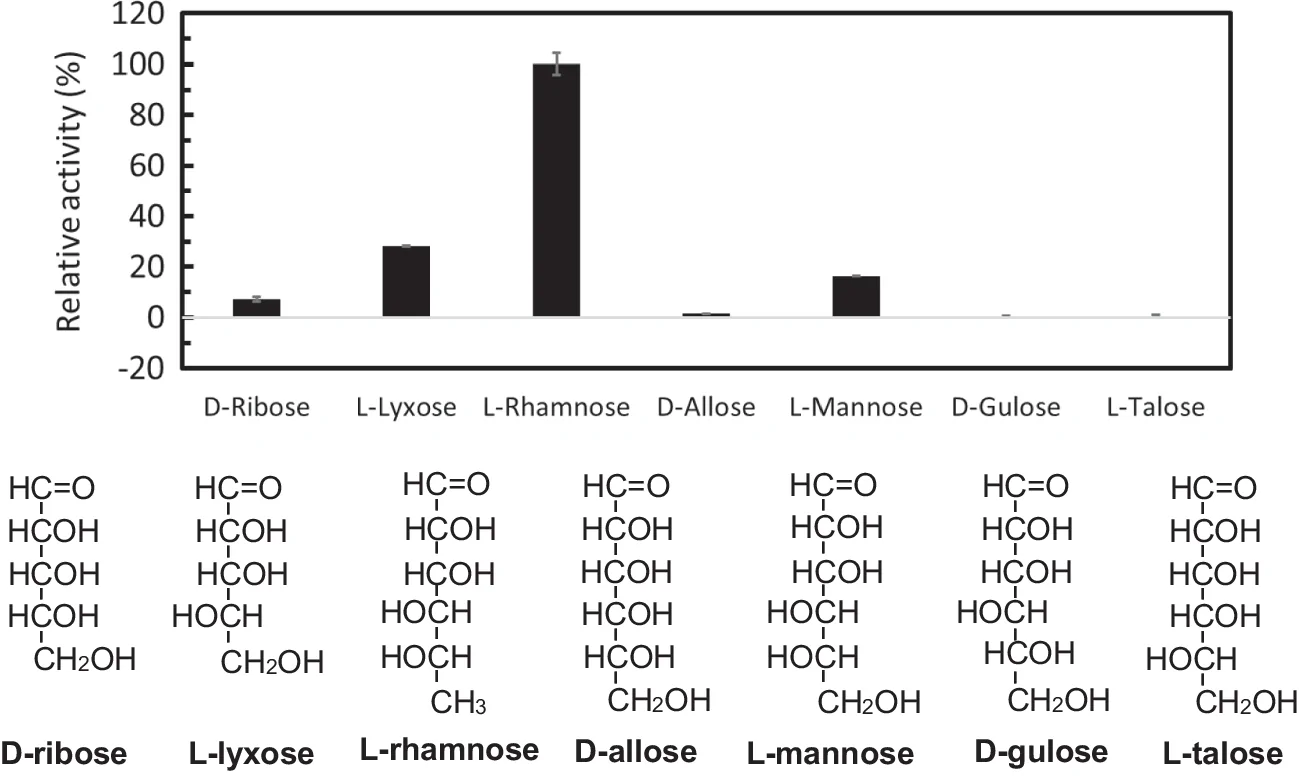
Substrate specificity of recombinant LrL-RhI. Substrate specificity of LrL-RhI was investigated using a sodium phosphate buffer (pH 5.5) containing 5 mM of each aldose (D-ribose, L-lyxose, L-rhamnose, D-allose, L-mannose, D-gulose, L-talose) and 1 mM CoCl_2_. The enzyme reaction was performed at 60 ℃. Each measurement was performed in triplicate

The highest activity of LrL-RhI was observed at pH 5.5 with a sodium phosphate buffer (Fig. 2a). An enzyme activity peak of LrL-RhI was observed at the reaction temperature of 70 °C (Fig. 2b); thus, the enzyme reaction was performed using 70 °C as the optimum reaction temperature for the analysis of pH stability and thermal stability (Fig. 2c, d). In the analysis of pH stability (Fig. 2c), high enzyme activity was retained at pH 3.6 and pH 9.0 after incubating LrL-RhI for 24 h at various pH levels. In the analysis of thermal stability (Fig. 2d), the enzyme activity after incubation at each temperature for 10 min was compared with the residual activity after incubation at 4 ℃ for 10 min as the control (100%). LrL-RhI was stable at a lower incubation temperature of 60 ℃, but the enzyme was almost inactivated after heat treatment at 70 ℃. Figure 2e shows the metal ion dependency of LrL-RhI. LrL-RhI did not reveal enzyme activity in the presence of EDTA alone, and metal ions were required, which showed the highest activity in the presence of CoCl_2_. Compared with the activity in the presence of CoCl_2_ (100%), the activity of LrL-RhI in the presence of MnCl_2_ was 37.7% and almost zero in the presence of other metal ions. To investigate the substrate specificity of LrL-RhI (Fig. 3), various aldoses were used, and LrL-RhI showed the highest enzyme activity toward L-rhamnose (173.1 U/mg, 100%), followed by L-lyxose (48.8 U/mg, 28.2%), L-mannose (28.3 U/mg, 16.4%), D-ribose (12.6 U/mg, 7.3%), and D-allose (2.5 U/mg, 1.5%). No activity was observed toward D-gulose and L-talose.

### Overall structure of LrL-RhI

Four crystal structures, such as LrL-RhI alone and complexes with L-rhamnose (LrL-RhI/L-rhamnose), D-allulose (LrL-RhI/D-allulose), and D-allose (LrL-RhI/D-allose), were determined at resolutions of 1.90, 1.61, 1.73, and 1.71 Å, respectively. All structures belong to the orthorhombic space group *P*2_1_2_1_2_1_ with four molecules (Mol-A, Mol-B, Mol-C, and Mol-D) forming a homotetramer in an asymmetric unit.

A part of the flexible loop region (Asp46-Thr73) was almost missing in the structures of LrL-RhI alone (missing Asn54-Ser64) and complexes with L-rhamnose (missing Pro53-Ser64) and D-allose (missing Asn54-Gly65) due to the weak electron density maps, but the corresponding region in the structure of LrL-RhI/D-allulose (Mol-A and Mol-B) was relatively visible, and a part of the region (Asn54-Gly65) was refined with 50% occupancy. The bound metal ions with strong electron density maps were refined as Mn^2+^ since LrL-RhI was expressed in cells in a medium containing 1 mM MnCl_2_.

**Fig. 4:**
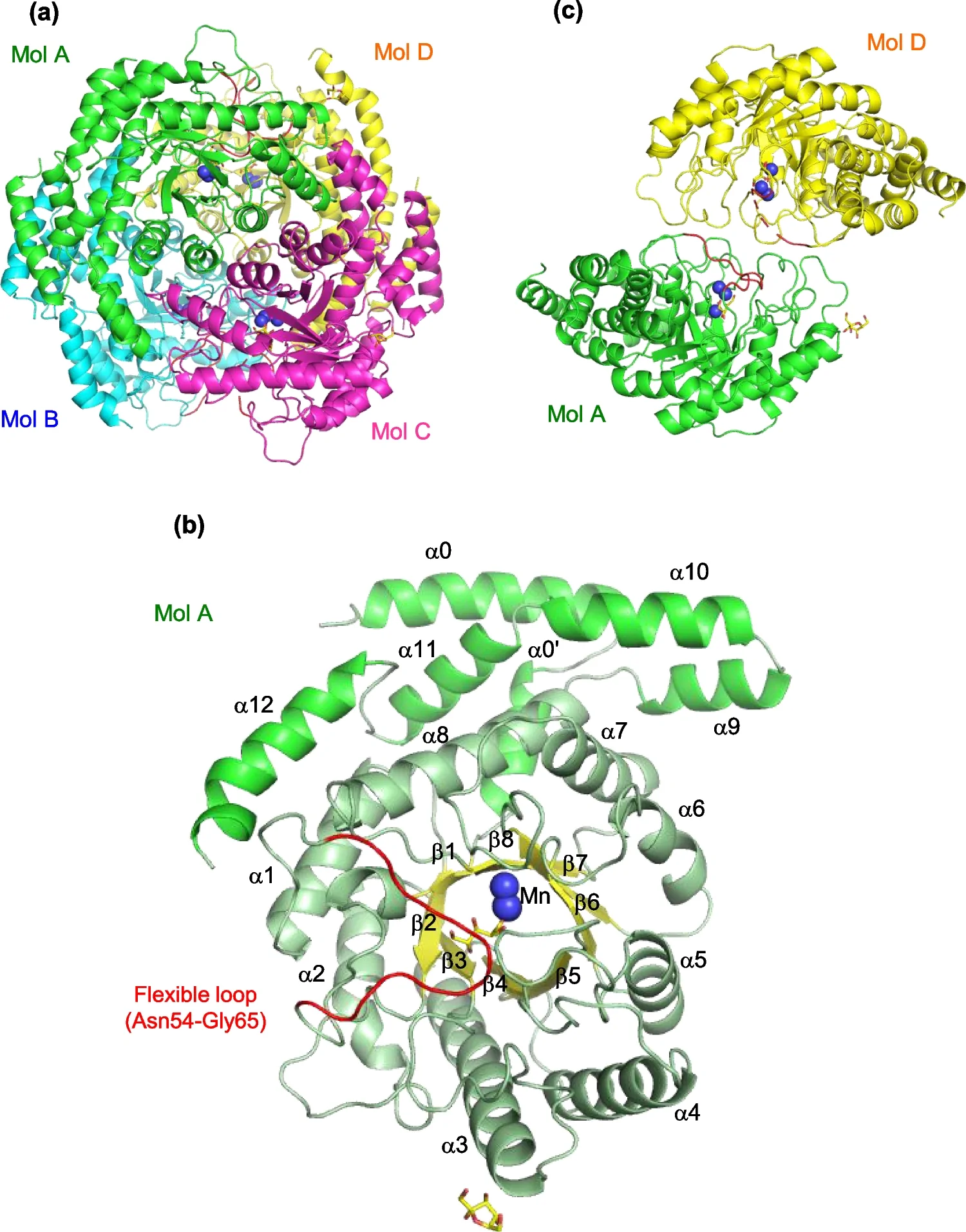
Overall structure of recombinant LrL-RhI. **a** Overall structure of homotetrameric LrL-RhI (labeled with Mol-A, Mol-B, Mol-C, and Mol-D) in the complex with D-allulose. **b** Structure of monomer of LrL-RhI (Mol-A) in the complex with D-allulose. The highly flexible loop region (Asn54-Gly64) is labeled. The bound D-allulose and metal ions are shown as sticks and spheres, respectively. **c** Dimeric structure of LrL-RhI (Mol-A and Mol-D). The dotted line is missing the flexible loop region in Mol-D

The overall structure of LrL-RhI/D-allulose is shown in Fig. 4a. Its structure is similar to the known structures of L-RhIs that form homotetramers. Each molecule adopts a (β/α)_8_ barrel fold with additional six α-helices (α0, α0′, α9, α10, α11, and α12), as shown in Fig. 4b, specifically for Mol-A of LrL-RhI/D-allulose. At the center of the beta barrel, there is an active site formed with two metal ions (structural metal and catalytic metal) and covered by a flexible loop region (Asn54-Gly65) like a lid, which is colored red. The flexible loop region is located between β1 and α1 (Asp46-Thr73). A ring-opened D-allulose (PSJ, yellow stick model) was bound in the active site, and an α-D-psicofuranose (PSV, yellow stick model) was found on the surface of Mol-A (near α3). The dimer form (Mol-A and Mol-D) is shown in Fig. 4c. The flexible loop region that is colored red (Asn54-Gly65) is visible in Mol-A, but the corresponding region with a red dotted line in Mol-D is missing (A56-V63) (Fig. 4c).

### Ligand-binding structure

#### Ligand-free and L-rhamnose

**Fig. 5:**
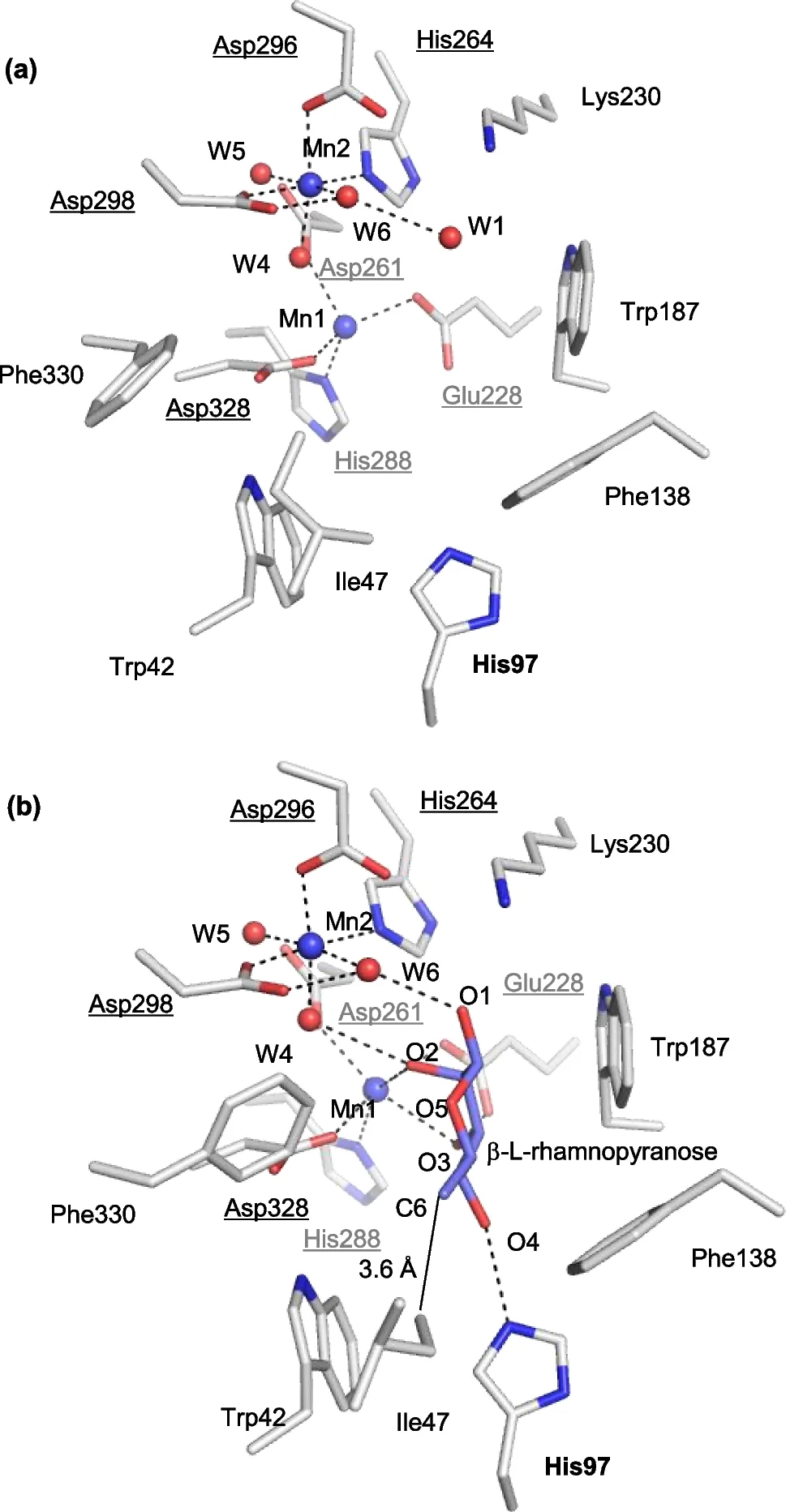

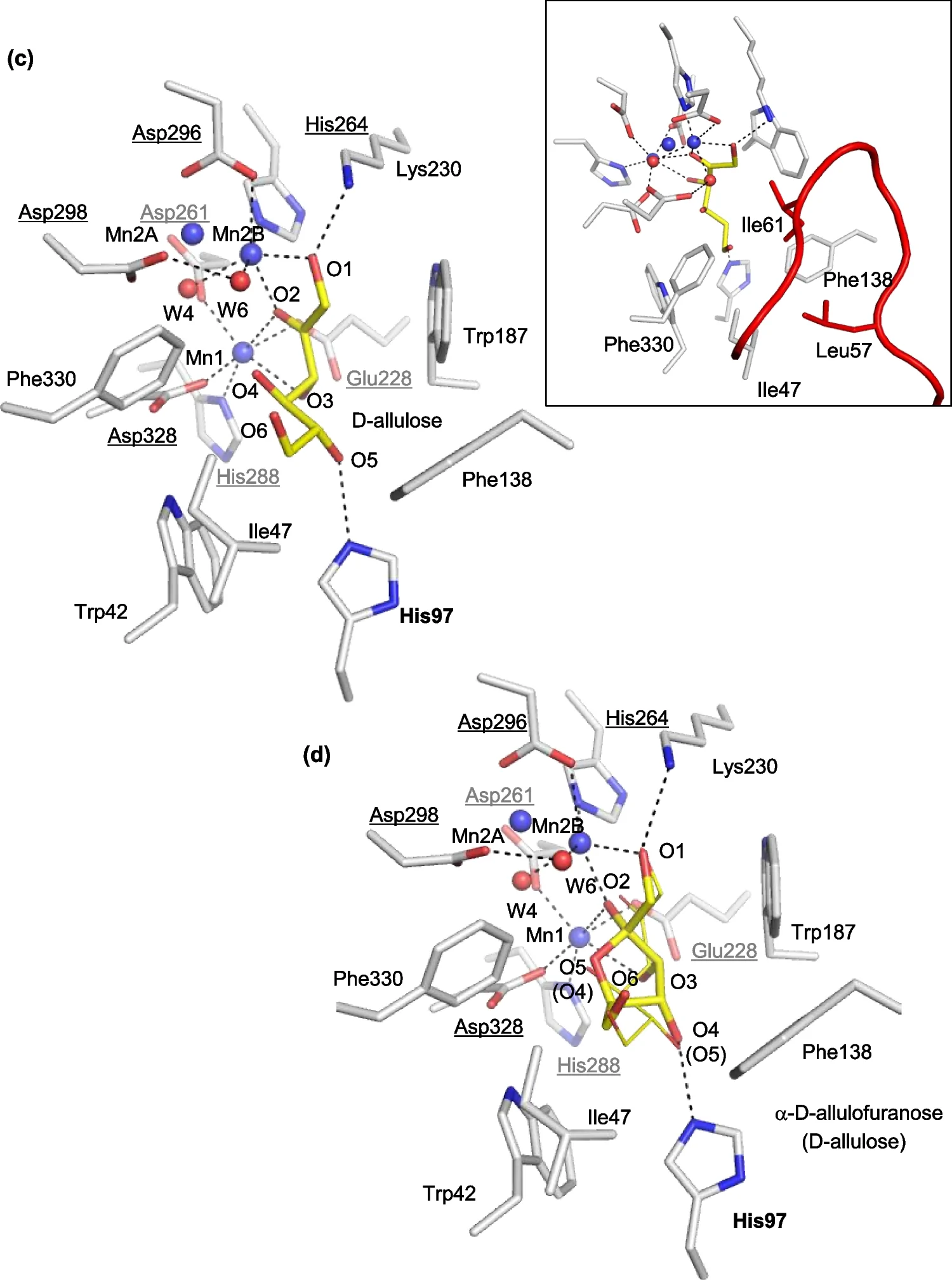

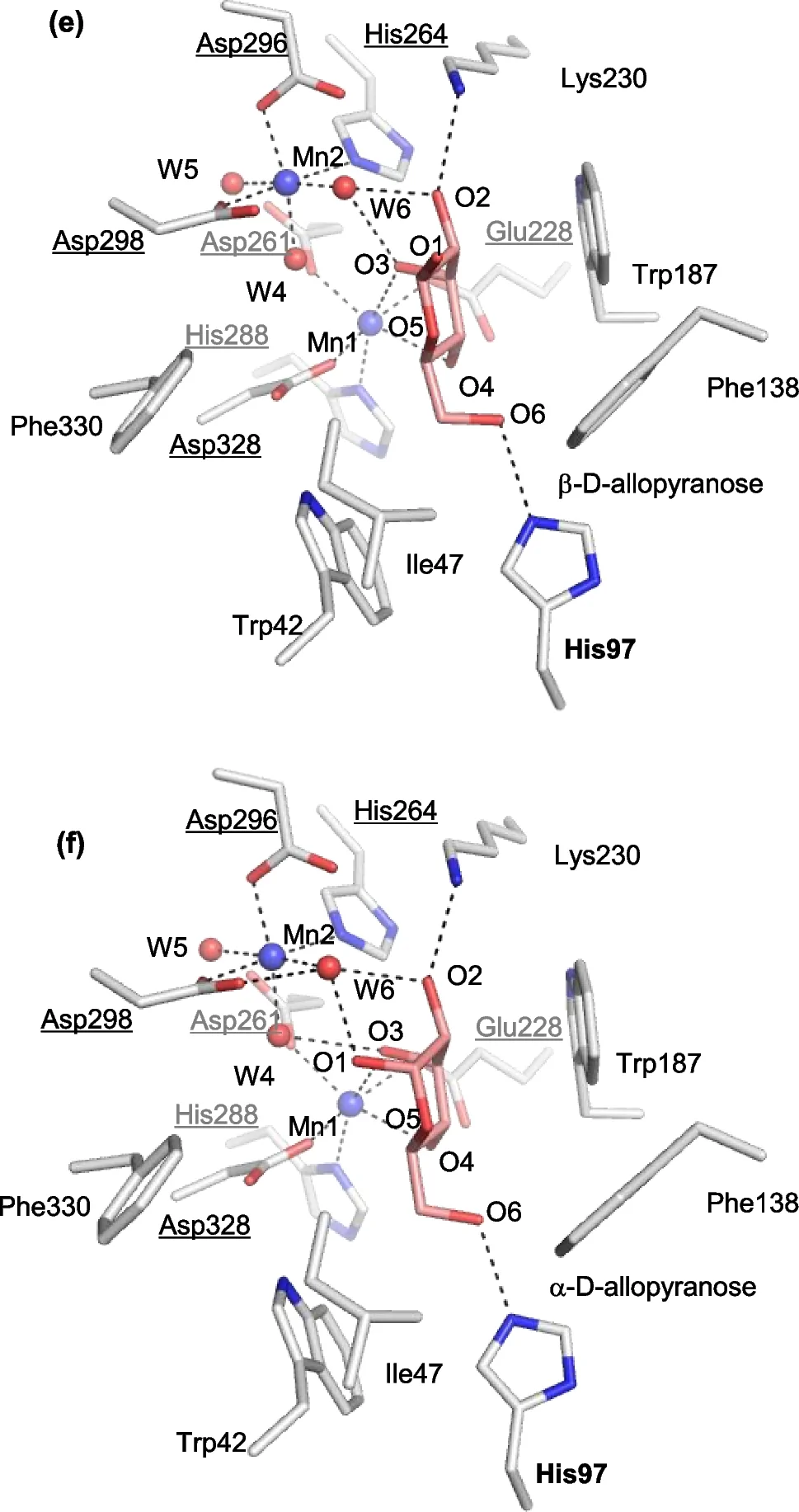
Substrate-binding site of recombinant LrL-RhI. **a** LrL-RhI (Mol-B) and **b** LrL-RhI/L-rhamnose (Mol-B). The manganese ions and water molecules are shown as spheres labeled with Mn and W, respectively. The bound L-rhamnopyranose is shown in a stick model with labels. The selected interactions among amino acid residues, metal ions, and water molecules are indicated by dotted lines. **c** LrL-RhI/D-allulose (Mol-B) and **d** LrL-RhI/D-allulose (Mol-C). The manganese ions and water molecules are shown as spheres labeled with Mn and W, respectively. The bound D-allulose and D-allulofuranose are shown in a stick and line model with labels. The labels in parentheses belong to the line model. The observed flexible loop is shown in a box at the top corner. The selected interactions among amino acid residues, metal ions, and water molecules are indicated by dotted lines. **e** LrL-RhI/D-allose (Mol-B) and **f** LrL-RhI/D-allose (Mol-D). The manganese ions and water molecules are shown as spheres labeled with Mn and W, respectively. The bound β-D-allopyranose and α-D-allopyranose are shown in a stick model with labels. The selected interactions among amino acid residues, metal ions, and water molecules are indicated by dotted lines

The structures of the catalytic site in the ligand-free structure (Mol-B of the structure of LrL-RhI) and the complex with L-rhamnose (β-L-rhamnopyranose) (Mol-B of the structure of LrL-RhI/L-rhamnose) are shown in Fig. 5a, b, respectively. The structural metal (Mn1) is coordinated by Glu228, Asp261, His288, and Asp328, and the catalytic metal (Mn2) is coordinated by His264, Asp296, Asp298, and three water molecules (W4, W5, and W6) (Fig. 5a). In the complex of LrL-RhI/L-rhamnose (Fig. 5b), Mn1 is coordinated by the same four residues (Glu228, Asp261, His288, and Asp328), as well as O2 and O3 of β-L-rhamnopyranose. The resulting coordination shows that Mn1 is stabilized in the catalytic site by the binding substrate. In contrast, Mn2 shows the same coordination as that of the ligand-free structure and is not coordinated with β-L-rhamnopyranose. Instead of the direct coordination with catalytic metal, O1 of β-L-rhamnopyranose forms a hydrogen bond with W6, which is coordinated with Mn2. O4 of β-L-rhamnopyranose is recognized by His97, and the hydrophobic side of pyranose is surrounded by hydrophobic residues (Trp187 and Phe138) and stabilized in the catalytic site, although part (Asn54-Ser64) of the flexible region is missing and does not cover the active site.

### D-allulose

In the LrL-RhI complex with D-allulose, a flexible loop region is relatively visible in Mol-A and Mol-B, but the Ala56-Val63 region is missing in Mol-C and Mol-D. Figure 5c shows the structure of the catalytic site of Mol-B in LrL-RhI/D-allulose. The bound D-allulose shows a ring-opened structure. The catalytic metal likely moves between two positions (Mn2A and Mn2B) and is refined as alternative metals Mn2A and Mn2B with 0.8 and 0.2 occupancies, respectively, considering the electron density maps (Supplemental Fig. S3b, S3c) and b-factors. D-allulose was refined with 1.0 occupancy. The catalytic metal at the position of Mn2B can be coordinated by His264 and Asp296, two water molecules (W4 and W6), and O1 and O2 of D-allulose. Mn1 is coordinated in the same manner in the structure of LrL-RhI/L-rhamnose, with four residues (Glu228, Asp261, His288, and Asp328) and O2 and O3 of D-allulose. O1 and O5 of D-allulose are recognized by Lys230 and His97, respectively, and Trp187 faces the hydrophobic side of D-allulose. In addition, Leu57 and Ile61 on the flexible loop region cover the active site and create a hydrophobic environment along with Phe138, Ile47, and Phe330 orienting to D-allulose.

Figure 5d shows the catalytic site of Mol-C in the structure of LrL-RhI/D-allulose. The flexible loop region (Ala56-Val63) is missing, and two positions of the catalytic metal are found and refined with 0.8 occupancy for Mn2A (the same position in Mol-B) and 0.2 occupancy for Mn2B. Considering the electron density maps for the bound D-allulose (Supplemental Fig. S3d), α-D-allulofuranose (before ring-opening, yellow stick model) and ring-opened D-allulose (yellow line model) were refined with 0.5 occupancy each. His97 recognizes O4 of α-D-allulofuranose instead of O5 of D-allulose.

### D-allose

Figure 5e shows the structure of the catalytic site of Mol-B in LrL-RhI/D-allose. The bound D-allose exhibits a pyranose form and appears to be β-D-allopyranose as revealed by the electron density maps (Supplemental Fig. S3e). Mn1 is coordinated by four residues (Glu228, Asp261, His288, and Asp328) and O3 and O4 of β-D-allopyranose. The coordination of the bound β-D-allopyranose in the catalytic site of LrL-RhI is different from the bound β-L-rhamnopyranose or α-D-allulofuranose in the catalytic site.

In the catalytic site of Mol-D in LrL-RhI/D-allose, D-allose may be bound as α-D-allopyranose as revealed by the electron density maps (Fig. 5f and Supplemental Fig. S3f).

## Discussion

The recombinant LrL-RhI showed the highest activity at pH 5.5 in the presence of MnCl_2_ (Fig. 2a), which was expected to be acidic because probiotic *Lactobacillus rhamnosus* Probio-M9 grows in acidic conditions. With the addition of CoCl_2_, a high activity was shown between pH 4.0 and pH 5.5 (Supplemental Fig. S4a). In the analysis of pH stability (Fig. 2c), an interesting result was observed. The optimum pH of LrL-RhI was pH 5.5 in the presence of MnCl_2_; however, high enzyme activity was retained at pH 3.6 and pH 9.0 after incubation for 24 h. Since both buffers (pH 3.6 and pH 9.0) are glycine-based, glycine may support the stability of LrL-RhI. To investigate the effect of glycine, the enzyme activity was compared after incubation for 24 h in a 20 mM sodium phosphate buffer (pH 7.0), a 20 mM glycine–HCl buffer (pH 3.6), and a 20 mM sodium phosphate buffer including 20 mM glycine (pH 7.0) (Supplemental Fig. S4c). There was no significant difference; however, the activity was slightly higher with the sodium phosphate/glycine buffer. The optimum reaction temperature of the recombinant LrL-RhI was 70 °C in the presence of MnCl_2_ (Fig. 2b) and CoCl_2_ (Supplemental Fig. S4b), which was not the optimum growth temperature of *L. rhamnosus* Probio-M9. Although it is unknown whether or not LrL-RhI is a constitutive or inducible enzyme in the strain, the recombinant LrL-RhI appears to be stabilized in a reaction buffer including 5 mM L-rhamnose and 1 mM MnCl_2_ (or 1 mM CoCl_2_). This is different from the in vivo condition, and the highest activity of the recombinant enzyme could be observed at a relatively higher temperature. Such a stabilization of the recombinant enzyme has been reported elsewhere (Yoshida et al. 2021). In the analysis of thermal stability (Fig. 2d), LrL-RhI was almost inactivated after heat treatment at 70 ℃ for 10 min. Although the peak of enzyme activity was 70 °C (Fig. 2b), the recombinant enzyme was not tolerant for a 10-min incubation at 70 °C. From the analysis of metal dependency, LrL-RhI showed the highest activity in the presence of CoCl_2_. The substrate specificity of LrL-RhI was investigated using a sodium phosphate buffer (pH 5.5) containing 5 mM of various aldoses and 1 mM of CoCl_2_. LrL-RhI showed the highest enzyme activity toward L-rhamnose, followed by L-lyxose, L-mannose, D-ribose, and D-allose. The activity toward D-allose was low, but it was recognized by LrL-RhI.

The overall structure of LrL-RhI is similar to the known structures of L-RhIs. The structurally similar proteins were L-RhI from *Escherichia coli* with L-rhamnose (EcL-RhI/L-rhamnose, PDB code 1de6, 51% identity, 0.9 Å r.m.s.d., 62.8 Z-score), L-RhI from *Bacillus halodurans* (BhL-RhI, 3p14, 51%, 0.9, 59.2), and L-RhI from *Pseudomonas stutzeri* (PsL-RhI, 2hcv, 18%, 2.1, 38.0), according to the Dali search (Holm and Laakso 2016; Holm 2022). At the dimer interface of LrL-RhI, the flexible loop region (Asn54-Gly65) covers the active site in its own molecule (Fig. 4b, c), as was observed in EcL-RhI (Korndörfer et al. 2000) (Supplemental Fig. S5a and S5b, the second figures to the left), although this corresponding loop region in PsL-RhI covers the active sites of each neighboring molecule at the dimer interface (Yoshida et al. 2007) (Supplemental Fig. S5a and S5b, right figures). This swapping mode for covering the active site at the dimer interface has been observed in D-xylose isomerases, and the movement of the flexible loop region of PsL-RhI is similar. In the case of BhL-RhI, the corresponding regions are missing in the structure (Supplemental Fig. S5a and S5b, the second figures to the right), and the activity at the dimer interface remains unknown. In this study, the flexible loop region of LrL-RhI was observed in the LrL-RhI/D-allulose complex; however, the corresponding regions in most of the LrL-RhI structures are also missing, suggesting that these regions are highly flexible. The corresponding loop regions in the complex of LrL-RhI/D-rhamnose are also missing (Supplemental Fig. S5a and S5b, left figures).

Supplemental Fig. S5c (upside) shows the surface model of L-RhIs with the electrostatic potential revealing positively and negatively charged areas, which are colored blue and red, respectively. A comparison of the surface model of LrL-RhI/L-rhamnose with those of EcL-RhI/L-rhamnose and BhL-RhI revealed a relatively strong red-colored passage from the surface to the active site in LrL-RhI/L-rhamnose. In addition, considering that BhL-RhI is missing a part of the flexible region, whole flexible loop regions (Asp46-Thr73 in LrL-RhI, Asp51-Arg78 in EcL-RhI, and Asp41-Thr68 in BhL-RhI) were deleted (Supplemental Fig. S5c) from each structure compared with the surface model. LrL-RhI showed a relatively strong red-colored area around the active site compared with the models of EcL-RhI and BhL-RhI. Although there is no large difference in the theoretical pI among four of the L-RhIs calculated based on amino acid sequence (LrL-RhI: 5.47; EcL-RhI: 5.48; BhL-RhI: 5.58; PsL-RhI:5.72), the optimal pH of 5.5 for LrL-RhI differs from the others, which have been reported as 7.6 for EcL-RhI (Takagi and Sawada 1964), 7.0 for BhL-RhI (Prabhu et al. 2011), and 9.0 for PsL-RhI (Bhuiyan et al. 1997; Leang et al. 2004a). The relatively strong negatively charged surface suggests that the optimum pH for the activity of LrL-RhI is lower and that the suitable condition is acidic.

In the complex of LrL-RhI/L-rhamnose (β-L-rhamnopyranose) (Fig. 5b), Mn2 shows the same coordination as that of the ligand-free structure (Fig. 5a), and it is not coordinated with β-L-rhamnopyranose. In the complex structure of PsL-RhI with L-rhamnose (Yoshida et al. 2007), Mn2 was found near the position of W6 observed in LrL-RhI and coordinated with O1 and O2 of L-rhamnose after ring-opening of β-L-rhamnopyranose, existing at the equilibrium of isomerization (Yoshida et al. 2007). Since catalytic metal is known to move between two positions in PsL-RhI (Yoshida et al. 2010b), the structure of the catalytic site of LrL-RhI/L-rhamnose probably shows the state before ring-opening of the substrate and isomerization.

Although a pH of 6.5 and manganese ions are not suitable for achieving the optimal enzyme activity of LrL-RhI, the crystal obtained under these conditions provided the structure before the ring-opening of the substrate. Comparing the ligand-free structure and the complex with L-rhamnose, the side chain of Phe330 away from the active site is oriented to C6 of β-L-rhamnopyranose in the structure of LrL-RhI/L-rhamnose. In addition, CD1 of visible Ile47 on the flexible region approaches C6 of β-L-rhamnopyranose, with a distance of 3.6 Å, although a part (Asn54-Ser64) of the flexible loop region is missing in both structures. A hydrophobic environment created by Trp42, Ile47, and Phe330 surrounds the methyl group at C6 of L-rhamnose and stabilizes the substrate binding. The highest enzyme activity of LrL-RhI is toward L-rhamnose, followed by L-lyxose, L-mannose, D-ribose, and D-allose. Unless the methyl group exists at C6 of L-rhamnose (L-lyxose), the ligand binding may be destabilized by the loss of hydrophobic interaction. In the case of L-mannose, the hydroxyl group at C6 may conflict with the hydrophobic environment or the size of the binding pocket.

In the LrL-RhI complex with D-allulose (Fig. 5c, d), the catalytic metal likely moves between two positions (Mn2A and Mn2B). In the structure of LrL-RhI/D-allulose (Fig. 5d), an alternative conformation of D-allulose was found, α-D-allulofuranose (before ring-opening, yellow stick model) and ring-opened D-allulose (yellow line model). His97 recognizes O4 of α-D-allulofuranose instead of O5 of D-allulose. The distance between W6 and O5 of α-D-allulofuranose is 3.2 Å, and the distance between W6 and O4 of D-allulose is 3.4 Å. Since the distance between W6 and O4 of D-allulose is 2.9 Å in the Mol-B, W6 might work as a catalytic water that moves cooperatively with the catalytic metal after the ring-opening of O5 of α-D-allulofuranose by catalytic Asp328 with a distance of 2.9 Å. After the ring-opening of α-D-allulofuranose, the distance between Asp328 and O4 of D-allulose is 3.1 Å in Mol-B. Thus, aldose-ketose isomerization may occur through a metal-mediated hydride-shift mechanism with the transfer of a proton between O1 and O2 by catalytic water. The catalytic water is activated as a hydroxide ion by the catalytic metal (Mn2B) (Korndörfer et al. 2000; Yoshida et al. 2007, 2010b, 2012). The distance between W6 and O1 of α-D-allulofuranose or D-allulose is 2.5 Å in Mol-C. The distance between W6 and O1 of D-allulose is 2.5 Å in Mol-A and Mol-B, 2.4 Å in Mol-D, and 2.5 Å for O1 of α-D-allulofuranose in Mol-D.

In the structure of LrL-RhI/D-allose, the bound D-allose exhibits a pyranose form and appears to be β-D-allopyranose (Fig. 5e) or α-D-allopyranose (Fig. 5f). Mn1 is coordinated by four residues (Glu228, Asp261, His288, and Asp328) and O3 and O4 of D-allopyranose. This binding ring-form may not be ring-opened or undergo isomerization. Even if the pyranose ring were opened, metal coordination with O1, O2, and O3 is required for aldose-ketose isomerization between D-allose and D-allulose by L-RhI. The coordination of the bound D-allopyranose in the catalytic site of LrL-RhI is different from the bound β-L-rhamnopyranose or α-D-allulofuranose in the catalytic site, and it appears to be bound in an inactive form. In the obtained structure, the crystal was soaked in a solution containing 30% (w/v) D-allose, which might be loosely recognized by LrL-RhI and work as an inhibitor in such a high concentration. However, in the LrL-RhI/D-allulose complex, ring-opened D-allulose is bound in the catalytic site, and D-allulose can be catalyzed in LrL-RhI. The enzyme activity of LrL-RhI toward D-allulose under the optimum condition was confirmed by high-performance liquid chromatography (Supplemental Fig. S6a). Therefore, D-allose could bind in the catalytic site of LrL-RhI, but it might be unfavorable when D-allose is used as the substrate at a high concentration under an unoptimized condition, and this may be the reason why the enzyme activity toward D-allose was low.

In this study, a recombinant LrL-RhI was expressed, and its crystal structures in complexes with L-rhamnose, D-allulose, and D-allose were determined. Although LrL-RhI may cause substrate/product inhibition under high concentrations of D-allose or under unoptimized conditions, LrL-RhI exhibited enzyme activity toward L-rhamnose, D-allulose, and D-allose under an acidic pH of 5.5. Information on the structure of LrL-RhI that shows activity under a low pH would be useful for pH-shift engineering of L-RhIs which work under basic conditions. Such an engineering would repress the non-enzymatic aldose-ketose isomerization which is caused under basic conditions.

## Supplementary Information

Below is the link to the electronic supplementary material.

Supplementary file1 (PDF 1805 KB)

Supplementary file2 (MTZ 9711 KB)

Supplementary file3 (PDB 2308 KB)

Supplementary file4 (MTZ 16046 KB)

Supplementary file5 (PDB 2459 KB)

Supplementary file6 (MTZ 12762 KB)

Supplementary file7 (PDB 2377 KB)

Supplementary file8 (MTZ 13332 KB)

Supplementary file9 (PDB 2364 KB)

## Data Availability

All data accompanying this research are presented directly in the manuscript and supplementary materials.

## References

[CR1] Bai W, Shen J, Zhu YM, Men Y, Sun YX, Ma YH (2015) Characteristics and kinetic properties of L-rhamnose isomerase from *Bacillus**subtilis* by isothermal titration calorimetry for the production of D-allose. Food Sci Technol Res 21:13–22. 10.3136/fstr.21.13

[CR2] Bhuiyan SH, Itami Y, Izumori K (1997) Isolation of an L-rhamnose isomerase-constitutive mutant of *Pseudomonas* sp. strain LL172: purification and characterization of the enzyme. J Ferment Bioeng 84:319–323. 10.1016/S0922-338X(97)89251-3

[CR3] Chen Z, Xu W, Zhang W, Zhang T, Jiang B, Mu W (2018a) Characterization of a thermostable recombinant L-rhamnose isomerase from *Caldicellulosiruptor**obsidiansis* OB47 and its application for the production of L-fructose and L-rhamnulose. J Sci Food Agric 98:2184–2193. 10.1002/jsfa.8703 28960307 10.1002/jsfa.8703

[CR4] Chen Z, Chen J, Zhang W, Zhang T, Guang C, Mu W (2018b) Improving thermostability and catalytic behavior of L-rhamnose isomerase from *Caldicellulosiruptor**obsidiansis* OB47 toward D-allulose by site-directed mutagenesis. J Agric Food Chem 66:12017–12024. 10.1021/acs.jafc.8b05107 30370768 10.1021/acs.jafc.8b05107

[CR5] Dische Z, Borenfreund A (1950) A spectrophotometric method for the microdetermination of hexosamines. J Biol Chem 184:517–522. 10.1016/S0021-9258(19)50982-6 15428432

[CR6] Duan S, Chen Y, Wang G, Li Z, Dong S, Wu Y, Wang Y, Ma C, Wang R (2023) A study of targeted mutation of L-rhamnose isomerase to improve the conversion efficiency of D-allose. Enz Microbial Technol 168:110259. 10.1016/j.enzmictec.2023.110259 10.1016/j.enzmictec.2023.110259 37245327

[CR7] Domagk GF, Zech R (1963) On the decomposition of desoxy sugars by bacterial enzymes. I. L-rhamnose-isomerase from *Lactobacillus**plantarum*. Biochem Z 339:145–153 14095156

[CR8] Emsley P, Lohkamp B, Scott W, Cowtan K (2010) Features and development of Coot. Acta Crystallogr D Biol Crystallogr 66:486–501. 10.1107/S0907444910007493 20383002 10.1107/S0907444910007493 PMC2852313

[CR9] Hayashi N, Iida T, Yamada T, Okuma K, Takehara I, Yamamoto T, Yamada K, Tokuda M (2010) Study on the postprandial blood glucose suppression effect of D-psicose in borderline diabetes and the safety of long-term ingestion by normal human subjects. Biosci Biotechnol Biochem 74:510–519. 10.1271/bbb.90707 20208358 10.1271/bbb.90707

[CR10] Heikkilä MP, Saris PEJ (2003) Inhibition of *Staphylococcus**aureus* by the commensal bacteria of human milk. J Appl Microbiol 95:471–478. 10.1046/j.1365-2672.2003.02002.x 12911694 10.1046/j.1365-2672.2003.02002.x

[CR11] Hill C, Guarner F, Reid G, Gibson GR, Merenstein DJ, Pot B, Morelli L, Canani RB, Flint HJ, Salminen S, Calder PC, Sanders ME (2014) The international scientific association for probiotics and prebiotics consensus statement on the scope and appropriate use of the term probiotic. Nat Rev Gastroenterol Hepatol 11:506–514. 10.1038/nrgastro.2014.66 24912386 10.1038/nrgastro.2014.66

[CR12] Holm L, Laakso LM (2016) Dali server update. Nucleic Acids Res 44:W351–W355. 10.1093/nar/gkw357 27131377 10.1093/nar/gkw357 PMC4987910

[CR13] Holm L (2022) Dali server: structural unification of protein families. Nucleic Acids Res 50:W210–W215. 10.1093/nar/gkac387 35610055 10.1093/nar/gkac387 PMC9252788

[CR14] Iida T, Yamada T, Hayashi N, Okuma K, Izumori K, Ishii R, Matsuo T (2013) Reduction of abdominal fat accumulation in rats by 8-week ingestion of a newly developed sweetener made from high fructose corn syrup. Food Chem 138:781–785. 10.1016/j.foodchem.2012.11.017 23411176 10.1016/j.foodchem.2012.11.017

[CR15] Kabsch W (2010) XDS. Acta Crystallogr D Biol Crystallogr 66:125–132. 10.1107/S0907444909047337 20124692 10.1107/S0907444909047337 PMC2815665

[CR16] Kim YS, Shin KC, Lim YR, Oh DK (2013) Characterization of a recombinant L-rhamnose isomerase from *Dictyoglomus**turgidum* and its application for L-rhamnulose production. Biotechnol Lett 35:259–264. 10.1007/s10529-012-1069-2 23070627 10.1007/s10529-012-1069-2

[CR17] Korndörfer IP, Fessner WD, Matthews BW (2000) The structure of rhamnose isomerase from *Escherichia**coli* and its relation with xylose isomerase illustrates a change between inter and intra-subunit complementation during evolution. J Mol Biol 300:917–933. 10.1006/jmbi.2000.3896 10891278 10.1006/jmbi.2000.3896

[CR18] Kozak K, Charbonneau D, Sanozky-Dawes R, Klaenhammer T (2015) Characterization of bacterial isolates from the microbiota of mothers’ breast milk and their infants. Gut Microbes 6:341–351. 10.1080/19490976.2015.1103425 26727418 10.1080/19490976.2015.1103425 PMC4826109

[CR19] Leang K, Takada G, Ishimura A, Okita M, Izumori K (2004a) Cloning, nucleotide sequence, and overexpression of the L-rhamnose isomerase gene from *Pseudomonas**stutzeri* in *Escherichia**coli*. Appl Environ Microbiol 70:3298–3304. 10.1128/AEM.70.6.3298-3304.2004 15184124 10.1128/AEM.70.6.3298-3304.2004 PMC427750

[CR20] Leang K, Takada G, Fukai Y, Morimoto K, Granstrom TB, Izumori K (2004b) Novel reactions of L-rhamnose isomerase from *Pseudomonas**stutzeri* and its relation with D-xylose isomerase via substrate specificity. Biochim Biophys Acta 1674:68–77. 10.1016/j.bbagen.2004.06.003 15342115 10.1016/j.bbagen.2004.06.003

[CR21] Lin CJ, Tseng WC, Lin TH, Liu SM, Tzou WS, Fang TY (2010) Characterization of a thermophilic L-rhamnose isomerase from *Thermoanaerobacterium**saccharolyticum* NTOU1. J Agric Food Chem 58:10431–10436. 10.1021/jf102063q 20822145 10.1021/jf102063q

[CR22] Lin CJ, Tseng WC, Fang TY (2011) Characterization of a thermophilic L-rhamnose isomerase from *Caldicellulosiruptor**saccharolyticus* ATCC 43494. J Agric Food Chem 59:8702–8708. 10.1021/jf201428b 21761877 10.1021/jf201428b

[CR23] Liu W, Chen M, Duo L, Wang J, Guo S, Sun H, Menghe B, Zhang H (2020) Characterization of potentially probiotic lactic acid bacteria and bifidobacteria isolated from human colostrum. J Dairy Sci 103:4013–4025. 10.3168/jds.2019-17602 32113772 10.3168/jds.2019-17602

[CR24] LoCascio RG, Niñonuevo MR, Kronewitter SR, Freeman SL, German JB, Lebrilla CB, Mills DA (2009) A versatile and scalable strategy for glycoprofiling bifidobacterial consumption of human milk oligosaccharides. Microb Biotechnol 2:333–342. 10.1111/j.1751-7915.2008.00072.x 21261928 10.1111/j.1751-7915.2008.00072.x PMC3815754

[CR25] Marcobal A, Barboza M, Froehlich JW, Block DE, German JB, Lebrilla CB, Mills DA (2010) Consumption of human milk oligosaccharides by gut-related microbes. J Agric Food Chem 58:5334–5340. 10.1021/jf9044205 20394371 10.1021/jf9044205 PMC2866150

[CR26] Martín R, Langa S, Reviriego C, Jimínez E, Marín ML, Xaus J, Fernández L, Rodríguez JM (2003) Human milk is a source of lactic acid bacteria for the infant gut. J Pediatr 143:754–758. 10.1016/j.jpeds.2003.09.028 14657823 10.1016/j.jpeds.2003.09.028

[CR27] Moralejo P, Egan SM, Hidalgo E, Aguilar J (1993) Sequencing and characterization of a gene cluster encoding the enzymes for l-rhamnose metabolism in *Escherichia**coli*. J Bacteriol 175:5585–5594. 10.1128/jb.175.17.5585-5594.1993 8396120 10.1128/jb.175.17.5585-5594.1993 PMC206615

[CR28] Murshudov GN, Vagin AA, Dodson EJ (1997) Refinement of macromolecular structures by the maximum-likelihood method. Acta Crystallogr D Biol Crystallogr 53:240–255. 10.1107/S0907444996012255 15299926 10.1107/S0907444996012255

[CR29] Murshudov GN, Skubák P, Lebedev AA, Pannu NS, Steiner RA, Nicholls RA, Winn MD, Long F, Vagin AA (2011) REFMAC5 for the refinement of macromolecular crystal structures. Acta Crystallogr D Biol Crystallogr 67:355–367. 10.1107/S0907444911001314 21460454 10.1107/S0907444911001314 PMC3069751

[CR30] Noguchi C, Kamitori K, Hossain A, Hoshikawa H, Katagi A, Dong Y, Sui L, Tokuda M, Yamaguchi F (2016) D-Allose inhibits cancer cell growth by reducing GLUT1 expression. Tohoku J Exp Med 238:131–141. 10.1620/tjem.238.131 26829886 10.1620/tjem.238.131

[CR31] Park CS, Yeom SJ, Lim YR, Kim YS, Oh DK (2010) Characterization of a recombinant thermostable L-rhamnose isomerase from *Thermotoga**maritima* ATCC 43589 and its application in the production of L-lyxose and L-mannose. Biotechnol Lett 32:1947–1953. 10.1007/s10529-010-0385-7 20809285 10.1007/s10529-010-0385-7

[CR32] Park CS (2014) Characterization of a recombinant L-rhamnose isomerase from *Bacillus**subtilis* and its application on production of L-lyxose and L-mannose. Biotechnol Bioproc Eng 19:18–25. 10.1007/s12257-013-0597-5

[CR33] Poonperm W, Takata G, Okada H, Morimoto K, Granström TB, Izumori K (2007) Cloning, sequencing, overexpression and characterization of L-rhamnose isomerase from *Bacillus**pallidus* Y25 for rare sugar production. Appl Microbiol Biotechnol 76:1297–1307. 10.1007/s00253-007-1109-3 17653540 10.1007/s00253-007-1109-3

[CR34] Power J (1967) The L-rhamnose genetic system in *Escherichia**coli* K-12. Genetics 55:557–568. 10.1093/genetics/55.3.557 5341476 10.1093/genetics/55.3.557 PMC1211410

[CR35] Prabhu P, Doan TT, Jeya M, Kang LW, Lee JK (2011) Cloning and characterization of a rhamnose isomerase from *Bacillus**halodurans*. Appl Microbiol Biotechnol 89:635–644. 10.1007/s00253-010-2844-4 20852996 10.1007/s00253-010-2844-4

[CR36] Prabhu P, Doan TN, Tiwari M, Singh R, Kim SC, Hong MK, Kang YC, Kang LW, Lee JK (2014) Structure-based studies on the metal binding of two-metal-dependent sugar isomerases. FEBS J 281:3446–3459. 10.1111/febs.12872 24925069 10.1111/febs.12872

[CR37] Ruibo X, Xu L, Weicheng L, Xin S, Heping Z (2023) Reveal the differences in the genome of *Lactobacillus**rhamnosus* Probio-M9 and other *Lactobacillus**rhamnosus* based on comparative genomics. J Chin Inst Food Sci Technol 23:254–264

[CR38] Ruiz L, García-Carral C, Rodriguez JM (2019) Unfolding the human milk microbiome landscape in the omics era. Front Microbiol 10:1378. 10.3389/fmicb.2019.01378 31293535 10.3389/fmicb.2019.01378 PMC6604669

[CR39] Sakwinska O, Moine D, Delley M, Combremont S, Rezzonico E, Descombes P, Vinyes-Pares G, Zhang Y, Wang P, Thakkar SK (2016) Microbiota in breast milk of Chinese lactating mothers. PLoS One 11:e0160856. 10.1371/journal.pone.0160856 27529821 10.1371/journal.pone.0160856 PMC4987007

[CR40] Seo MJ, Choi JH, Kang SH, Shin KC, Oh DK (2018) Characterization of L-rhamnose isomerase from *Clostridium**stercorarium* and its application to the production of D-allose from D-allulose (D-psicose). Biotechnol Lett 40:325–334. 10.1007/s10529-017-2468-1 29124517 10.1007/s10529-017-2468-1

[CR41] Shintani H, Shintani T, Sato M (2020) D-Allose, a trace component in human serum, and its pharmaceutical applicability. Int J Appl Biol 11:200–213

[CR42] Sun Y, Hayakawa S, Puangmanee S, Izumori K (2006) Chemical properties and antioxidative activity of glycated α-lactalbumin with a rare sugar, D-allose, by Maillard reaction. Food Chem 95:509–517. 10.1016/j.foodchem.2005.01.033

[CR43] Takagi Y, Sawada H (1964) The metabolism of L-rhamnose in *Escherichia**coli*. Biochim Biophys Acta 92:10–17. 10.1016/0926-6569(64)90264-0 14243758 10.1016/0926-6569(64)90263-9

[CR44] Takata G, Uechi K, Taniguchi E, Kanbara Y, Yoshihara A, Morimoto K, Izumori K (2011) Characterization of Mesorhizobium loti L-rhamnose isomerase and its application to L-talose production. Biosci Biotechnol Biochem 75:1006–1009. 10.1271/bbb.110018 21597169 10.1271/bbb.110018

[CR45] Tseng WC, Chen YC, Chang HC, Lin CJ, Fang TY (2022) Altering the substrate specificity of recombinant L-rhamnose isomerase from *Thermoanaerobacterium**saccharolyticum* NTOU1 to favour D-allose production. J Biotechnol 358:9–16. 10.1016/j.jbiotec.2022.08.015 36030895 10.1016/j.jbiotec.2022.08.015

[CR46] Vagin A, Teplyakov A (1997) MOLREP: An automated program for molecular replacement. J Appl Cryst 30:1022–1025. 10.1107/S0021889897006766

[CR47] Vagin A, Teplyakov A (2010) Molecular replacement with MOLREP. Acta Crystallogr D Biol Crystallogr 66:22–25. 10.1107/S0907444909042589 20057045 10.1107/S0907444909042589

[CR48] Wilson DM, Ajl S (1957) Metabolism of l-rhamnose by *Escherichia**coli*. I L-Rhamnose Isomerase J Bacteriol 73:410–414. 10.1128/jb.73.3.410-414.1957 13416204 10.1128/jb.73.3.410-414.1957 PMC289813

[CR49] Winn MD, Ballard CC, Cowtan KD, Dodson EJ, Emsley P, Evans PR, Keegan RM, Krissinel EB, Leslie AGW, McCoy A, McNicholas SJ, Murshudov GN, Pannu NS, Potterton EA, Powell HR, Read RJ, Vagin A, Wilson KS (2011) Overview of the CCP4 suite and current developments. Acta Crystallogr D Biol Crystallogr 67:235–242. 10.1107/S0907444910045749 21460441 10.1107/S0907444910045749 PMC3069738

[CR50] Yoshida H, Yamada M, Ohyama Y, Takada G, Izumori K, Kamitori S (2007) The structures of l-rhamnose isomerase from *Pseudomonas**stutzeri* in complexes with l-rhamnose and d-allose provide insights into broad substrate specificity. J Mol Biol 365:1505–1516. 10.1016/j.jmb.2006.11.004 17141803 10.1016/j.jmb.2006.11.004

[CR51] Yoshida H, Takeda K, Izumori K, Kamitori S (2010a) Elucidation of the role of Ser329 and the C-terminal region in the catalytic activity of *Pseudomonas**stutzeri* L-rhamnose isomerase. Protein Eng Des Sel 23:919–927. 10.1093/protein/gzq077 20977999 10.1093/protein/gzq077

[CR52] Yoshida H, Yamaji M, Ishii T, Izumori K, Kamitori S (2010b) Catalytic reaction mechanism of *Pseudomonas**stutzeri* L-rhamnose isomerase deduced from X-ray structures. FEBS J 277:1045–1057. 10.1111/j.1742-4658.2009.07548.x 20088877 10.1111/j.1742-4658.2009.07548.x

[CR53] Yoshida H, Yoshihara A, Teraoka M, Yamashita S, Izumori K, Kamitori S (2012) Structure of l-rhamnose isomerase in complex with l-rhamnopyranose demonstrates the sugar-ring opening mechanism and the role of a substrate sub-binding site. FEBS Open Bio 3:35–40. 10.1016/j.fob.2012.11.008 23772372 10.1016/j.fob.2012.11.008 PMC3668531

[CR54] Yoshida H, Yoshihara A, Kato S, Mochizuki S, Akimitsu K, Izumori K, Kamitori S (2021) Crystal structure of a novel homodimeric l-ribulose 3-epimerase from Methylomonus sp. FEBS Open Bio 11:1621–1637. 10.1002/2211-5463.13159 33838083 10.1002/2211-5463.13159 PMC8167858

