# Supplementary material for: X-ray structure and characterization of a probiotic Lactobacillus rhamnosus Probio-M9 L-rhamnose isomerase
Source: Appl Microbiol Biotechnol. 2024 Mar 2;108:249. doi: 10.1007/s00253-024-13075-9 (PMC10908623; doi:10.1007/s00253-024-13075-9)
Supplement: Supplementary file 1 [file 253_2024_13075_MOESM1_ESM.pdf]

Supplemental Information

X-ray structure and characterization of a probiotic *Lactobacillus rhamnosus* Probio-M9 L-rhamnose isomerase

Hiromi Yoshida[1,2*], Naho Yamamoto[3], Lin Hai Kurahara[4], Ken Izumori[2,3], Akihide Yoshihara[2,3]

ATGGTAAAACCTGAAGAGGTGGACAAAGCGTATGAAGTGGCCAAACAGCGCTACGCAGA
GATTGGAGTCGACACTGATGCAGCCATGAAAGAATTAGAGAAAGTACCGCTGAGTGTGCA
TTGTTGGCAAGGCGACGATATTCACGGTTTTCTGTTTCCGAATCAGGAATTAACAGGCGGC
ATTGGGGTGTCAGGCAACTATCCGGGCATTGCTCGTACTCCGGATGAATTAGCCGGTGATA
TGCACGAAGCACTCTCTCTGATCCCCGGTAAGCATCGCGTTCAGCTGCATGCGATTTATGC
CGTAACCGACAAGAAACGCGATTTGGACACCCTGGAACCGGAAGATTTCGACTACTGGAT
CGATTGGGCTAAACAGGAAGGAGTTGGCTTGGATTTTAACGGTACCTTCTTTTCGCATCCG
ATGGTGAAAGATAACATGACGGTCAGCTCTCCGGATCCCAAAGTCCGCGACTTTTGGATTC
GGCACGGCAAAATCTCTCGCGAAATTAGCAATTACATCGGTGAGAAACTGGGCAGCCAAG
TCGTGAATAACTTTTGGCTTCCTGATGGCTTCAAAGACAACCCGATCGATAAGAAAACCCC
TCGCTTGCGCTTACTGAAAGCCCTCGATGAAATCATCAAAGACCCACTCCCCGAGAAGAA
CACCATTGAGAGCTTTGAAGGGAAACTGTTTGGTACAGGCATTGAATCGTATACCACCGG
AAGTCACGAATTCTACCAGAATTACGCGATTTCCCGCAACAAGCTGTGGACGATTGATGCG
GGTCATTTTCATCCGACCGAAGATGTGTCCGATAAATTTAGCGCTTTCTTCCCGTTCGGTAA
AGGGCTGTTTATGCACGTGTCCCGTCCAGTACGTTGGGACTCAGATCATGTCGTGATCATG
GATGATGCGCTTATCCGCATTACGCGTAGCCTGGTTCGTGATGGCTATCTGGATCGGACGCA
TATTGGCCTGGATTTCTTTGACGCGACGATCAATCGTGTTGCGGCTTGGGTTGTTGGTGCC
CGTGCCACACAGAAAAGTCTGCTTCAAGCAATGCTGGCGCCAATTGACCAACTGAAGAA
AGATGAACTCAATGCGGATTTCACCACTCGCCTGATCGAAACGGAGGAATTGAAATCGTT
CCCGTTTGGTGCAGTCTGGGACAAATTTTGCCAAGATCACAATACTCCAGTGGGGTTCGA
CTGGATGAACAACATTCATCAGTATGAGAAAGACGTTCAGTTTAAACGTGATGCAAAGCT
GGTGCAC

Supplemental Fig. S1 Nucleotide sequence of synthesized gene of LrL-RhI

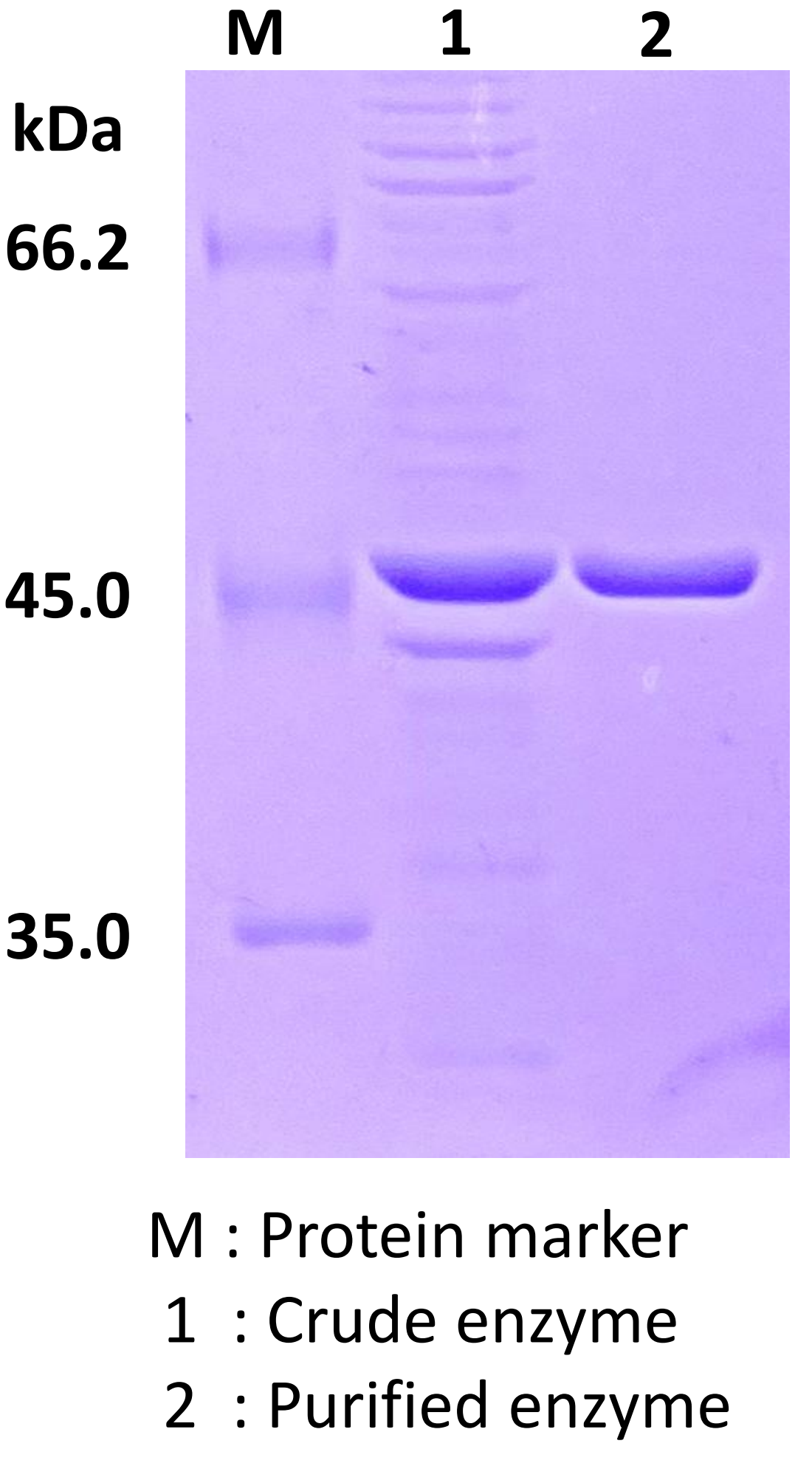

Supplemental Fig. S2 SDS-PAGE analysis of purification of recombinant LrL-RhI. The samples of the crude enzyme (lane1) and purified enzyme (lane2) are shown with protein markers.

Purification of LrL-RhI

	Total protein (mg)	Total activity (U)	Specific activity (U/mg)	Purification fold	Yield (%)
Crude enzyme	280	55.8	0.199	1.00	100
Purified enzyme	7.79	9.09	1.17	5.85	16.3

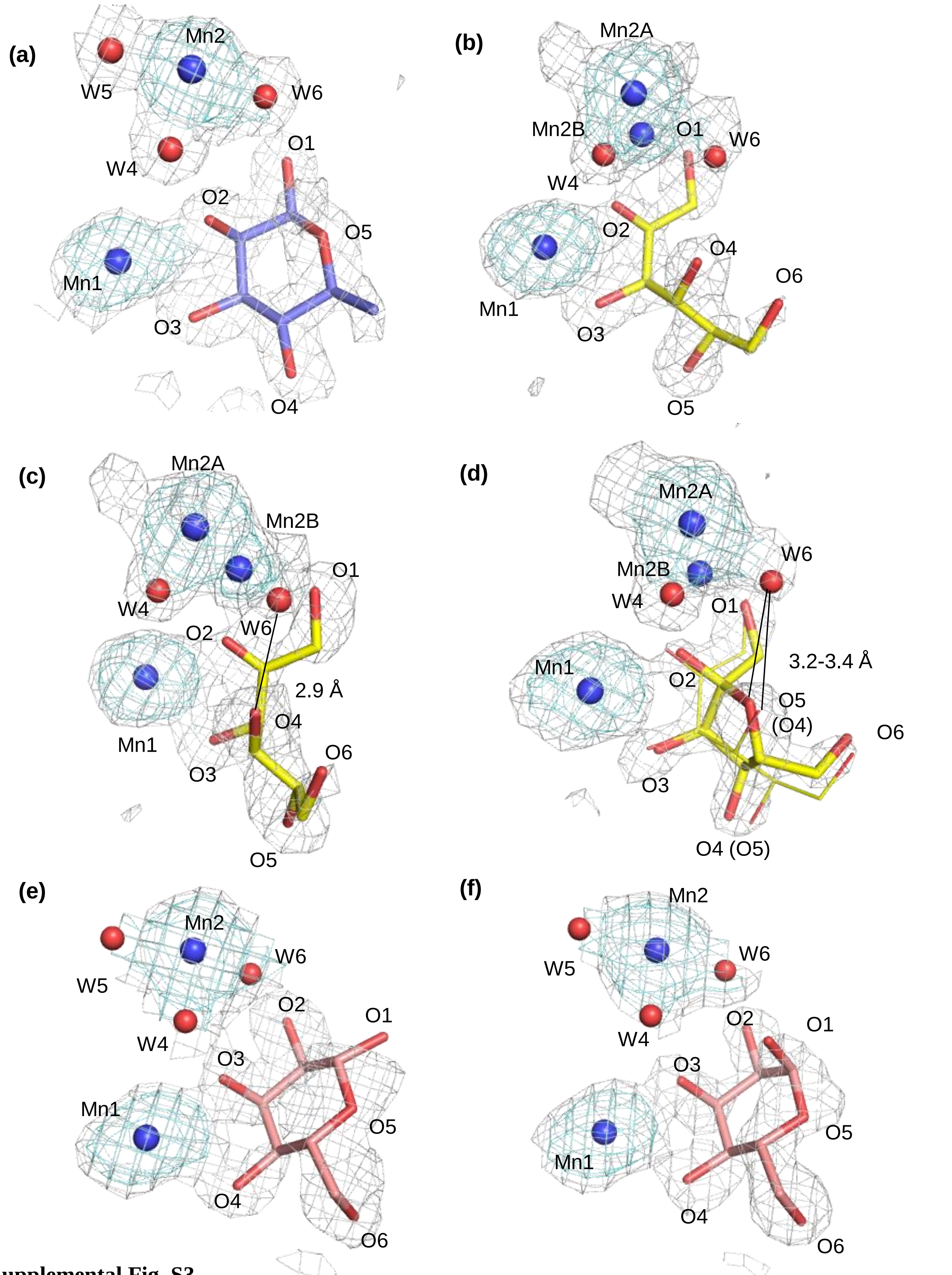

Supplemental Fig. S3

Metal ions, water molecules, and bound substrate with simulated annealing omit maps.

(a) LrL-RhI/L-rhamnose (Mol-B) with sa-omit maps at contour level 5.0 (cyan) and 3.0 (gray). (b, c) LrL-RhI/D-allulose (Mol-B) and (d) LrL-RhI/D-allulose (Mol-C) with sa-omit maps at contour level 5.0 (cyan) and 3.0 (gray). (e)LrL-RhI/D-allose (Mol-B) and (f) LrL-RhI/D-allose (Mol-D) with sa-omit maps at contour levels 5.0 (cyan) and 2.5 (gray).

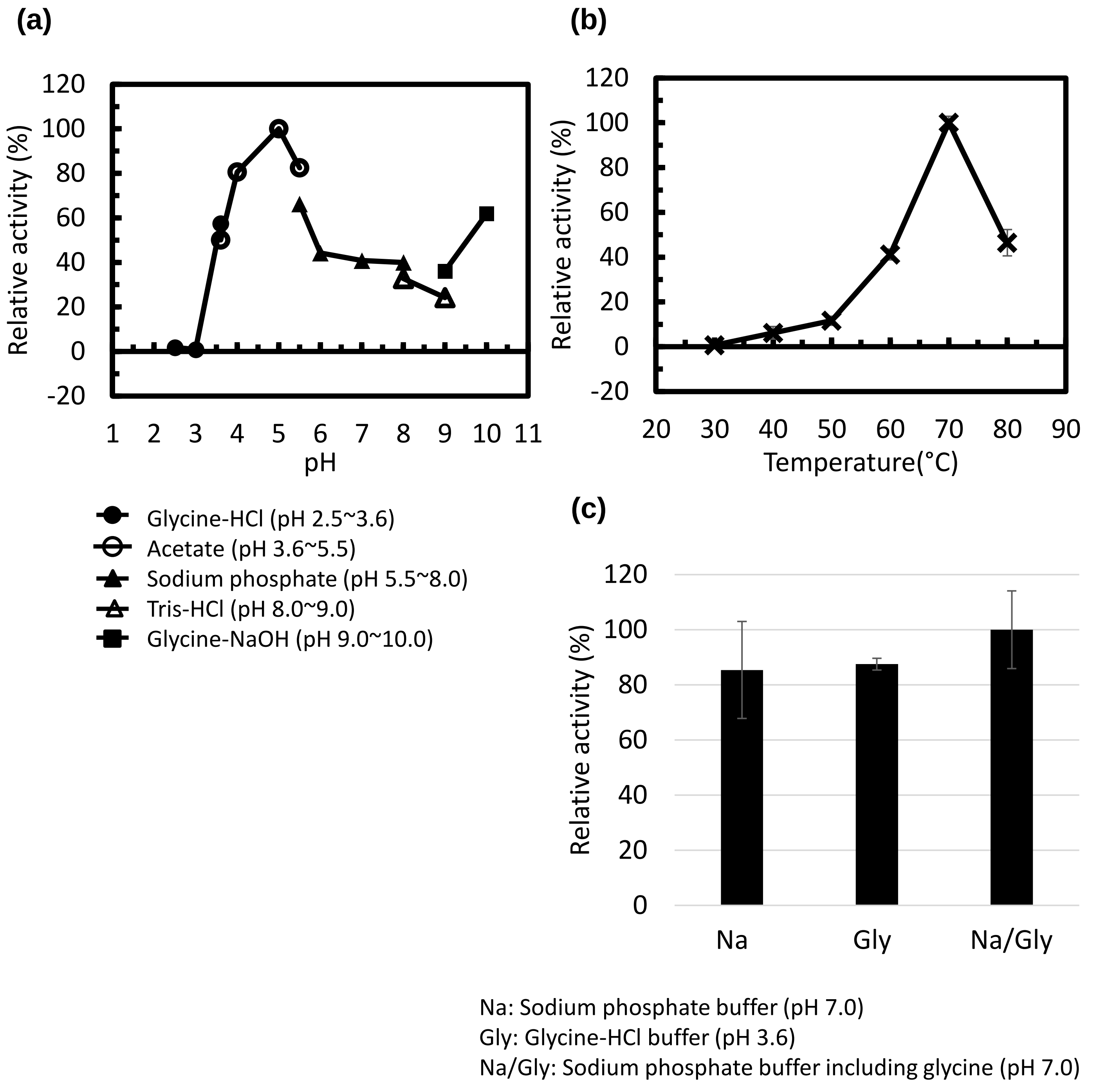

Supplemental Fig. S4 Enzyme properties of recombinant LrL-RhI in the presence of $CoCl_2$.
Effects of (a) pH on the enzyme activity, (b) temperature on the enzyme activity, and (c) additional glycine on the enzyme stability in the presence of $CoCl_2$.
To investigate the effects of pH (a), enzyme activity was measured using the following buffers: glycine-HCl buffer (pH 2.5–3.6, circles), acetate buffer (pH 3.6–5.5, open circles), sodium phosphate buffer (pH 5.5–8.0, triangles), Tris-HCl buffer (pH 8.0–9.0, open triangles), and glycine-NaOH buffer (pH 9.0–10.0, squares). The optimum pH was shown between 4.5 and 5.5. To investigate the effect of temperature (b), enzyme activity was measured after incubation at each temperature for 10 min using a sodium phosphate buffer (pH 5.5). The highest enzyme activity was observed at 70 ℃, which is the same as shown in the presence of $MnCl_2$. To investigate the effect of glycine for stability (c), the enzyme was incubated at 4 ℃ for 24 hours in a 20 mM sodium phosphate buffer (pH 7.0), a 20 mM glycine-HCl (pH 3.6), and a 20 mM sodium phosphate buffer including 20 mM glycine (pH 7.0). The remaining activity was measured using a sodium phosphate buffer (pH 5.5), containing 5 mM L-rhamnose and 1 mM $CoCl_2$, and the relative activity was determined. Slightly higher activity was shown in the sodium phosphate/glycine buffer. Each measurement was performed in triplicate.

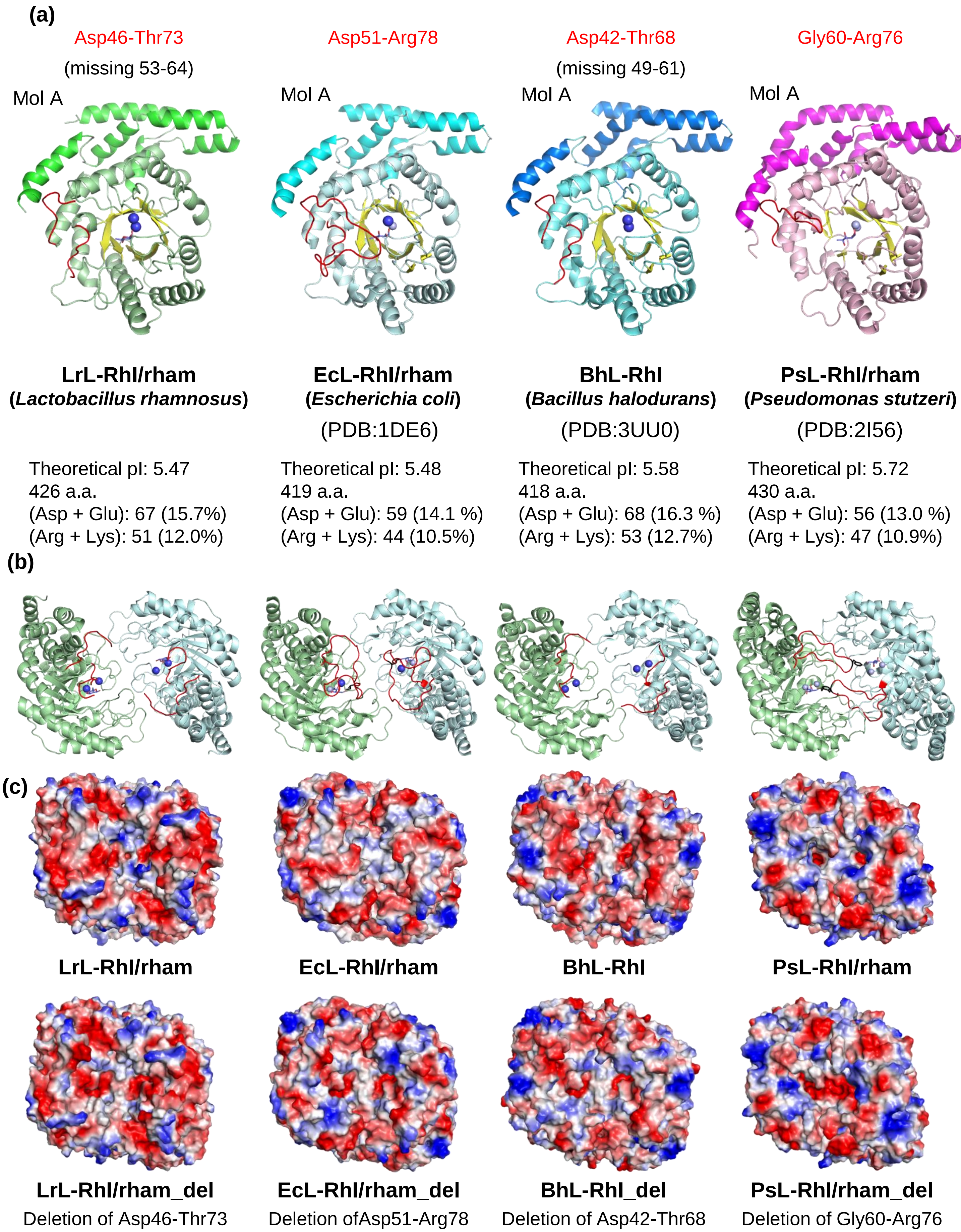

Supplemental Fig. S5

Comparison of monomer and dimer interfaces of L-RhIs. The flexible loop regions are colored red. The view of the surface model with electrostatics of L-RhIs is shown with positively and negatively charged areas, which are colored blue and red, respectively. In LrL-RhI, negatively charged areas are concentrated toward the active site. It may be a reason why the optimum pH for the activity of LrL-RhI is acidic.

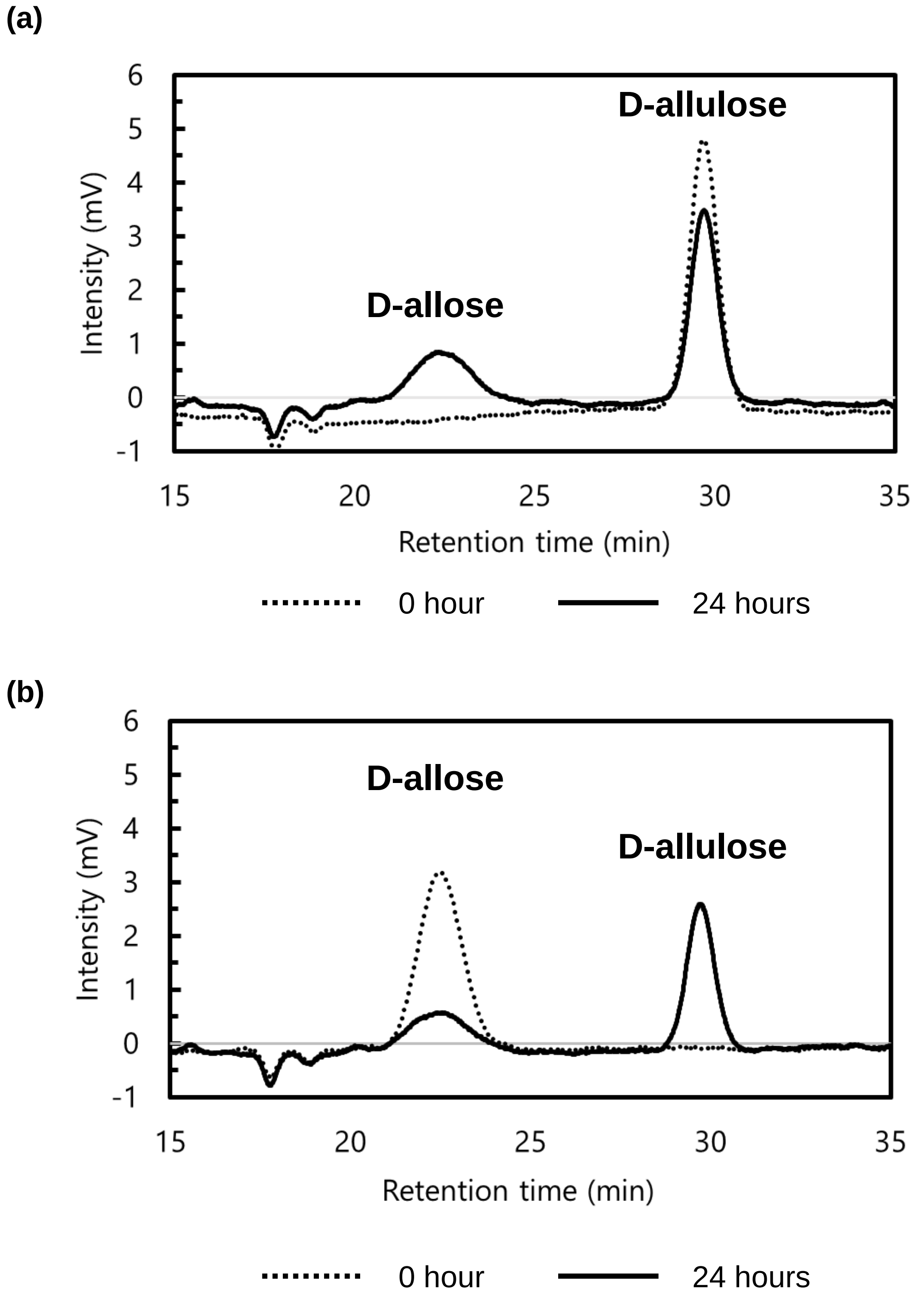

Supplemental Fig. S6

HPLC analysis of the production of D-allose and D-allulose by LrL-RhI.

(a) D-allose production from D-allulose and (b) D-allulose production from D-allose.

Purified LrL-RhI was reacted for 24 hours in a 35 mM sodium phosphate buffer (pH 5.5) containing 1 mM $CoCl_2$ and 5 mM substrate ((a) D-allulose or (b) D-allose). For HPLC analysis, a Shimadzu RID-20A refractive index detector and a Hitachi HPLC column GL-C611 were used. Separation was performed at 60ºC using 0.1 mM NaOH as an eluent at a flow rate of 1.0 mL/min. The results of the HPLC analysis are shown with a solid line (24 hours) and a dotted line (0 hour). The retention times of D-allose and D-allulose are 22.5 and 29.7 min, respectively.
